# The significance of neural inter-frequency power correlations

**DOI:** 10.1038/s41598-021-02277-0

**Published:** 2021-11-30

**Authors:** Oscar W. Savolainen

**Affiliations:** Centre for Bio-Inspired Technology, Imperial College London, London, UK

**Keywords:** Bioinformatics, Statistical methods

## Abstract

It is of great interest in neuroscience to determine what frequency bands in the brain have covarying power. This would help us robustly identify the frequency signatures of neural processes. However to date, to the best of the author’s knowledge, a comprehensive statistical approach to this question that accounts for intra-frequency autocorrelation, frequency-domain oversampling, and multiple testing under dependency has not been undertaken. As such, this work presents a novel statistical significance test for correlated power across frequency bands for a broad class of non-stationary time series. It is validated on synthetic data. It is then used to test all of the inter-frequency power correlations between 0.2 and 8500 Hz in continuous intracortical extracellular neural recordings in Macaque M1, using a very large, publicly available dataset. The recordings were Current Source Density referenced and were recorded with a Utah array. The results support previous results in the literature that show that neural processes in M1 have power signatures across a very broad range of frequency bands. In particular, the power in LFP frequency bands as low as 20 Hz was found to almost always be statistically significantly correlated to the power in kHz frequency ranges. It is proposed that this test can also be used to discover the superimposed frequency domain signatures of all the neural processes in a neural signal, allowing us to identify every interesting neural frequency band.

## Introduction

### Previous work on neural inter-frequency relationships

It is somewhat unclear in the literature what frequency bands in neural signals have covarying power. If the power in one frequency band goes up, does it also go up in another one, and for which frequency band pairs is this the case? This is an important question, as it has consequences for many subfields of neuroscience. These include modelling, understanding biological mechanisms, computational neuroscience, functional connectivity, the compression of neural signals in Brain–Machine Interfaces (BMI), and many more. Many studies have looked at inter-frequency relationships in neural signals. In general, they belong to one of two classes. The first consists of indirect decoding methods. The second involves directly comparing neural features.

#### Class 1: Indirect decoding methods

In this class, various neural time-frequency features are compared in their ability to decode various behaviours/stimuli. This class primarily serves to inform us about useful neural features for decoding. However, it has also historically stood as the basis for determining whether neural features contain common information.

To give a very few, non-exhaustive examples:^1,2^ investigated the ability of different Local Field Potential (LFP) power bands to decode various movements. In Ref.^3^, they decoded a hand-reaching task using different time-frequency features of intracortical neural signals, e.g. LFP, Single-Unit Activity (SUA), MUA, Entire Spiking Activity (ESA), using linear and deep learning methods. References^4–9^ compared the ability of spikes and LFPs to decode various behaviors using linear decoders. References^10–12^ compared SUA’s and LFP’s ability to decode a stimuli. References^4,13^ performed Feature of Importance (FOI) analysis on neural time-frequency features to determine their respective behavioral decoding performances.

Unless care is taken, the results from Class 1 are susceptible to misinterpretation. Orthogonal features may have similar decoding ability on even specific tasks, despite the features containing no common information. For example, how windy it is and whether it is raining both tell us about the weather, but they may be uncorrelated. As such, methodologically, the comparative decoding ability of different features does not always tell us about their common informational content. This can only be corrected by comparing the features between themselves, as is done in the next class.

#### Class 2: Direct comparison of neural features

The second class of studies involves direct comparison between various neural features. A non-exhaustive list includes:

In Ref.^14–16^, spike and LFP coherency during reaching tasks was examined. Reference^17^ investigated the spike-triggered average LFP (stLFP) in humans and non-human primates during undirected behavior. References^18–22^ looked at the relationship between SUA and LFP during stimuli. References^23,24^ respectively decoded multi-unit and single-unit spike trains from LFPs. In Refs.^25,26^, gamma-band LFPs were found to be significantly coupled to MUA activity on the same electrode. Reference^22^ measured the inter-frequency power correlations between different frequency bands in stLFPs. Reference^27^ looked at phase-coupling between LFP frequency bands and neural firing times. Reference^28^ discussed the relationship between the fMRI BOLD signal, SUA, MUA and LFPs. Reference^29^ reviewed what we know about the origin of extracellular fields and currents. The sub-field of Functional Connectivity has also looked extensively at inter-frequency relationships and how they vary across distance^30–36^.

However, there are some difficulties associated with getting a systematic understanding of neural inter-frequency power relationships, even from Class 2 studies. The first reason is because the subset of Class 2 studies that look at inter-frequency power relationships is smaller than the entire set of Class 2 studies. Many Class 2 studies look at other inter-frequency relationships, e.g. phase–amplitude relationships. It warrants mentioning that phase–amplitude relationships, for example, observe a non-linear relationship. They are interesting in that the amplitude of different frequencies can be modulated by the phase of another frequency band^37,38^. In contrast, inter-frequency power relationships are linear in nature, and can show if neural process have power signatures in multiple frequency bands. As such, although many other inter-frequency relationships exist to study, we think inter-frequency power correlations are a potentially interesting one that is worth looking at.

The second difficulty with interpreting the literature is that different studies regularly use different species, individuals, electrode arrays, electrode placements, electrode referencing schemes, attention loads, behaviours, stimuli, definitions, and feature extraction methods^4^. They also tend to focus on specific inter-frequency power relationships, e.g. one study looks at frequencies *a* and *b*, and another looks at frequencies *x* and *y*. A way to mitigate this last problem is that all of the inter-frequency power relationships in the signal can be tested simultaneously. That is the approach taken in this work, although it does not solve all of the other inter-study comparison problems that are present.

A third reason, as has been discussed elsewhere^39–42^, is that some neuroscientific studies do not account for autocorrelations in time-series data. Not doing so can result in spurious correlations^8,40–46^. It has been hypothesised that a lack of accounting for autocorrelation, or equivalently assuming independence of time-series features, has created spurious results in the sub-fields of Neuro-Imaging^40^ and Functional Connectivity^41^. As such, it may be contributing to a replication crisis in neuroscience. Related to this problem is the use of statistical tests designed for stationary data, e.g. the Fisher Z and Rayleigh tests, on non-stationary neural signals. The non-stationarity introduces autocorrelation within the power of different frequency bands, i.e. intra-frequency autocorrelation. This autocorrelation must also be accounted for.

A fourth reason is that some studies do not control the multiple testing problem under dependency. When the multiple testing problem is not controlled for, there can be a large chance of rejecting true null hypotheses^47^. Additionally, if the test statistics are dependent on each other, this can skew the results if not accounted for^47^.

A last reason is that some studies, when they use Time-Frequency Distributions (TFD), do not account for introduced biases. A large range of TFDs, specifically non-dyadic TFDs, oversample the frequency domain^48^. This means that nearby frequency bands in these TFDs will be found to contain some covarying power, regardless of the input signal. This is because they are partly measuring the same signal. In these cases, covaring power will be detected regardless of whether the components in the frequency bands that are not shared contain covarying power or not. As such, some bias is introduced in the case of nearby frequency bands by frequency-domain oversampled TFDs.

### The contribution of this work

It would likely be useful to gain insight into what frequency bands in neural signals have correlated power. This can help us identify the frequency-domain signatures of neural processes. To get a systematic understanding of inter-frequency power relationships, it would also likely be helpful to robustly analyse all present inter-frequency power relationships simultaneously in a dataset. Firstly, this makes it easier to compare results across different studies. Secondly, this may also help us to discover all of the present interesting inter-frequency power correlations in the dataset. This can mitigate the effects of selection bias in the literature, i.e. keep it from biasing towards a narrow selection of inter-frequency power correlations that are a priori believed to be potentially interesting and are investigated individually. Thirdly, in “Do only correlated frequency bands contain interesting information?” we hypothesize that if a frequency band does not have a significant power correlation to another frequency band, that indicates that it is likely not a signature of any neural process measured in the signal. As such, this test may enable us to discover every interesting frequency band in the neural signal.

In this work, we propose a novel Monte Carlo (MC) statistical significance test for the multiple testing of correlated power across frequency bands for a broad class of non-stationary time-series. This test controls the False Discovery Rate under a wide range of dependencies, and accounts for oversampling of the frequency domain and for intra-frequency autocorrelation. Specifically, we propose a statistical significance test for inter-scale power correlations in the Wavelet Power Spectrum (WPS) of the Continuous Wavelet Transform (CWT). To the best of the author’s knowledge, this method applies to any non-stationary time series under certain Dirichlet, non-monotonous and dependency conditions, discussed in “Signal conditions for the use of this test”. This method can be generalised to other TFDs, e.g. the Spectrogram, assuming they have an equal amount of points in the time-domain for all frequencies, discussed in “Choice of the continuous wavelet transform”. Finally, it can also be generalised to different measures of association, given certain normality constraints under the null, discussed in “Multiple testing of correlation matrices”.

We use this test to determine statistically significant inter-frequency power correlations in broadband intracortical neural signals from Macaque M1 cortex.

It warrants mentioning that^49^ proposed an analytic method for determining the significance of inter-frequency power correlations, used in that work for testing EEG data. The method assumes that the data fits a modulated cyclostationary model, which is a specific subset of non-stationarity signals. As such, in cases of modulated cyclostationary data with strong harmonics, the author recommends that the method from^49^ be used due to its analytical nature. However, for a broad class of non-stationary signals defined in “Signal conditions for the use of this test”, the method proposed in this work will work as it makes few assumptions about the data.

### The continuous wavelet transform

#### Choice of time-frequency decomposition

Neural signals are non-stationary^50^, and so classic Fourier decomposition is insufficient for their analysis. TFDs can be used to analyse how the power in different frequency bands in a signal changes over time. This allows one to determine what frequency bands contain covarying power.

Wavelet Transforms (WT) have been widely adopted in biomedical signal processing, including in neural data processing^51–61^. This is because they produce less distortion and have useful de-noising properties relative to bandpass filters^53,62,63^. WTs have even been compared with various other TFDs for neural data analysis^64^, and found to be competitive. The statistical properties of WTs have also been analysed in many different circumstances^45,48,65–69^. Therefore we chose to use WTs for our TFD. However, other TFDs can also be valid choices^64^.

#### Wavelet decomposition

A wavelet function, i.e. wavelet, is any function $\psi$ $\epsilon$ $\mathbf {L^{2}}$ $({\mathbb {R}})$ of zero average, $\vert \vert \psi \vert \vert$ = 1, and centered at *t* = 0^70^. By scaling, i.e. stretching, the mother wavelet $\psi$ by a positive quantity *a* and translating it by $\tau$ $\epsilon$ $({\mathbb {R}})$, we define a family of time-frequency atoms, $\psi _{a,\tau }$ as:

1 $$\begin{aligned} \psi _{a,\tau } = \frac{1}{\sqrt{a}} \, \psi \left( \frac{t-\tau }{a}\right) . \end{aligned}$$

Decomposing the time series signal along the time-frequency atoms produces a TFD. Scales correspond to frequencies and the translation gives us insight into the time domain^70^. $a \, \{a \; \epsilon \; \mathbb {N^+} | \; 1 \le a \le u \}$ is the scale index, where *u* is the number of analysed scales, i.e. frequency bands. In this work, the term scale is used to refer to frequency bands measured by the WT. This is further discussed in “Continuous wavelet decomposition and the morse wavelet”.

#### Choice of the continuous wavelet transform

The details of the WT decomposition depend on the orthogonality and scale ‘dyadicity’ of the WT in question^48^. Non-orthogonal WTs oversample the time domain, meaning that adjacent coefficients in the same scale can sample some of the same data. A non-orthogonal wavelet can be visualised as a window that moves one sample each time step, and uses the windowed samples to calculate a coefficient. Wider windows produce more autocorrelation between adjacent coefficients, as they largely sample the same data. As such, non-orthogonal WTs produce intra-scale autocorrelation, and the wider the scale the more autocorrelation. Inversely, orthogonal WTs optimally sample the time domain, so that the autocorrelation between adjacent intra-scale coefficients is approximately zero^48,71,72^. Therefore, in orthogonal WTs, wider scales produce fewer coefficients than narrower scales. This is because there is no overlap between the time samples contributing to each coefficient within each scale. E.g., if a scale was equal in width to half the signal length, an orthogonal sampling would only produce 2 samples at that scale. In this work, wavelet non-orthogonality will be referred to as time-domain oversampling, because it is more descriptive.

Scale non-dyadicity means that adjacent scales overlap somewhat. In other words, the frequency domain is over-sampled. This creates an intrinsic correlation between nearby scales regardless of the input signal. For example, the 5–10 Hz power band would seem to be correlated with the 8–13 Hz power band. Part of this correlation would be because they both sample power between 8–10 Hz. This is sometimes referred to as redundancy between scales. Dyadicity means that there is no overlap between scales: the frequency domain is optimally sampled. In this work scale non-dyadicity will be referred to as frequency-domain oversampling.

The Continuous Wavelet Transform (CWT) oversamples both time and frequency domains. The Discrete Wavelet Transform (DWT) optimally samples both domains. The Maximal Overlap DWT (MODWT), i.e. Stationary Wavelet Transform (SWT) optimally samples the frequency domain but oversamples the time domain^48,51^.

In our application, time-domain oversampling is a requirement. This is because calculating the Inter-Scale Power Correlation (ISPC) requires an equal number of coefficients per scale, which is not the case with optimally time-domain sampled scales. As such, only the CWT and MODWT are appropriate.

Between the CWT and MODWT, one would expect the MODWT to be more convenient for testing ISPCs, due to the optimal sampling of the frequency-domain. However, based on our observation of MODWT and CWT inter-scale power correlation matrices of intracortical neural data, the frequency oversampling of the CWT appears very valuable in terms of both interpretability and detail. As such, this work presents an inter-scale power correlation statistical significance test for CWT.

### Measuring inter-scale power correlations

**Figure 1:**
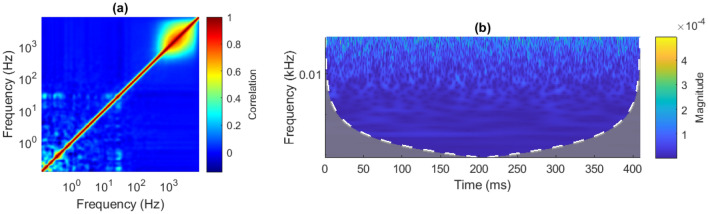
(**a**) Inter-Scale Power Correlation Matrix (ISPCM) *r* derived from neural recording Session  1, Chan. 1. The frequency axes are on a logarithmic scale. (**b**) Wavelet Power Spectrum (WPS) of a short segment of Session  1, Chan. 1.

To measure the CWT ISPCs in a signal, first one takes the CWT of the analysed signal. Then one obtains the WPS by taking the point-wise square of the CWT’s absolute value. In this work, the neural WPS is denoted as **S**, where individual scales are denoted as $\mathbf {s_a}$, where $a \, \{a \; \epsilon \; \mathbb {N^+} | \; 1 \le a \le u \}$ is the scale index, and *u* is the number of analysed scales. Next, one takes the time-wise correlation between WPS scales $\mathbf {s_a}$ and $\mathbf {s_b}$, for all $1 \le a,b \le u$ pairs. This gives the 2D Inter-Scale Power Correlation Matrix (ISPCM) of the analysed signal, of size $u \times u$. An example WPS and ISPCM from the neural data analysed in this work is given in Fig. 1.

In this work, the Morse wavelet is used. As such, the ISPCM can be interpreted as the inter-frequency power correlation matrix, due to the unique mapping of scale to frequency for the Morse wavelet^73^. Taking the ISPCM tells one how correlated the power in different frequency bands is, given the CWT decomposition. This process is extensively discussed in the “Methods” section.

### Multiple testing of correlation matrices

In this work, we need to perform multiple statistical tests simultaneously. Specifically, we need to test all of the different elements of the neural ISPCM. However, taking the test results to be statistically significant based on their unadjusted marginal *p*-values creates a large risk of rejecting true null hypotheses^47^. This is generally solved by controlling the expected number of falsely rejected hypotheses, the FDR, at some proportion $\alpha$, with $0 \le \alpha \le 1$^74^.

Reference^75^ pioneered work in demonstrating that strong dependencies between test statistics, such as those intrinsic to a correlation matrix, can significantly degrade the effectiveness of many FDR procedures. In Ref.^76^, Cai and Liu proposed a method for testing each element of a correlation matrix that successfully controls the FDR under a wide range of dependencies. This method has 2 parts. The first step transforms the to-be-tested Pearson correlation values $r_{a,b}$ into test statistics, $T_{a,b}$. $T_{a,b}$ needs to be asymptotically normally distributed under the null. The second step performs multiple testing on $T_{a,b}$ while controlling the multiplicity under dependency.

For this work, we used MC processes to produce normal, null hypothesis, ISPC distributions. We then tested which elements of the neural ISPCMs were significantly different from the nulls, using the second step from Ref.^76^. We were not able to use the test statistic proposed in Ref.^76^. This was due to the significant intra-scale autocorrelation in our signals, which violated i.i.d assumptions.

### A Monte Carlo test statistic

**Figure 2:**
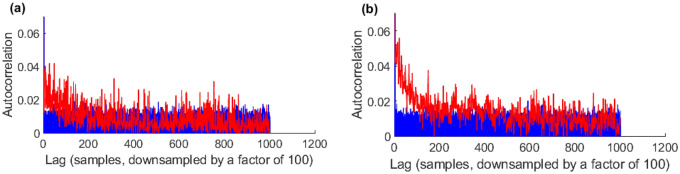
Plots demonstrating examples of the intra-scale autocorrelation of non-stationary pre-whitened neural signals vs. that of stationary white noise. Red shows the intra-scale autocorrelation function of neural data, Session 1, Chan. 1. Blue represents the intra-scale autocorrelation functions of 100 white noise processes of the same length as the neural recording. (**a**) Scale 20, i.e. a central frequency of 1.6 kHz. (**b**) Scale 40, i.e. a central frequency of 570 Hz.

When testing for significant ISPCs between time and frequency oversampled scales in non-stationary signals, three sources of spurious correlation need to be accounted for. The first is the ISPC due to the frequency-domain oversampling. This is produced by the TFD, independent of the input signal. This is where adjacent scales partially sample the same frequencies, which creates an intrinsic correlation between the adjacent scales, discussed earlier in “Choice of the continuous wavelet transform”. The second source of bias is the intra-scale autocorrelation introduced by the TFD’s oversampling of the time-domain. This is where adjacent WPS coefficients in the same scale can be significantly autocorrelated, also discussed in “Choice of the continuous wavelet transform”. If autocorrelation is not accounted for while determining the significance of cross-correlations, this can produce falsely significant results^8,40–46^. The third source of bias is the intra-scale autocorrelation due to 2nd order properties of the non-stationary signal. In a non-stationary signal, the autocorrelation structure of the power in a frequency band may be significantly different from that predicted by stationary white noise input to the TFD. This can be observed in Fig. 2.

We want to know if the observed neural ISPCs are significant or not. As such, we need to test the neural ISPCs against an appropriate null distribution. Our null distribution, which accounts for all three sources of spurious ISPC, is the sum of 2 elements. The first is the mean intrinsic CWT Morse wavelet ISPCM from stationary white noise input. With white noise input, the power in frequency bands is non-autocorrelated. As such, any measured ISPC will be due to the other two sources of bias. The first is the frequency-domain oversampling of the CWT. The second is random chance when taking the cross-correlation between two autocorrelated sequences, where the autocorrelation is due only to the CWT time-domain oversampling. However, the mean cross-correlation between two separately autocorrelated, otherwise random sequences is zero. As such, across many white noise processes, the mean white noise ISPCM estimates the contribution of only the frequency-domain oversampling. This is a ‘distorting’ contribution of the CWT, independent of the input signal. An example of the mean ISPCM from white noise processes of length 500 s can be seen in the Supplementary Information, Fig. 12a,b.

The second element is a normal distribution, and depends on the input signal. It is obtained by taking the neural WPS **S**, and phase randomising each scale $\mathbf {s_{a}}$ independently. Phase-randomisation is a common method to find the significance of correlations between autocorrelated time-series^8,41,43–46^. We then calculated the ISPCM of the phase-randomised WPS ${\mathbf {S}}$. We repeated this multiple times for each recording in a MC simulation. The resulting distribution of ISPCs across MC iterations originates purely from intra-scale autocorrelation. This intra-scale autocorrelation is due to both the CWT time-domain oversampling and the non-stationarity of the signal. The phase-randomisation destroys the ISPC due to CWT frequency-domain oversampling, as any shared inter-scale phase relation is randomised.

For a neural ISPC to be significant, it needs to be statistically unlikely for it to originate from the combination of the three spurious sources. Therefore, we test whether the neural ISPC is larger than that predicted by the sum of the two MC-generated elements, while controlling the multiplicity with the procedure from^76^. As such, any significant deviation from the null statistically represents a genuine ISPC in the tested signal, rather than a spurious contribution of the CWT and signal intra-frequency autocorrelation. See Methods “CWT inter-scale power correlation significance test implementation” for details on the production of the null distributions, and on multiple testing using the method from Ref.^76^. The motivation for adding the two elements to create the null distribution is also discussed further in “Conservatively removing the effects of the frequency-domain oversampling”.

In short, we obtained the distribution of inter-frequency cross-correlations due to auto-correlations from phase-randomised frequency bands. We then controlled the multiplicity of the problem, while testing if the observed neural inter-frequency power correlations were significantly different to those in the phase-randomised data. This is more or less what one would expect from a correlation test between multiple autocorrelated sequences: account for the autocorrelation, account for the multiplicity. However, an additional component is included, the mean white noise ISPCM, which controls for the bias of the TFD itself. It shows how correlated different frequency bands are because of the frequency domain oversampling of the TFD, not because of the signal. One can think of it as finding the distortion in the lens we use to observe the true ISPCM. By inputting frequency bands that have uncorrelated power, we can see how the TFD affects the measured ISPCM. Similarly, inputting straight light beams through a lens would show how the light is being bent, allowing one to measure the distortion caused by the lens. A MODWT would in theory not have any distortion because the frequency domain is not oversampled, but the tradeoff is that one loses out on frequency resolution. As such, the final test looks at the significance of the correlations once the autocorrelation, frequency domain oversampling, and multiplicity have been controlled for.

## Results

**Figure 3:**
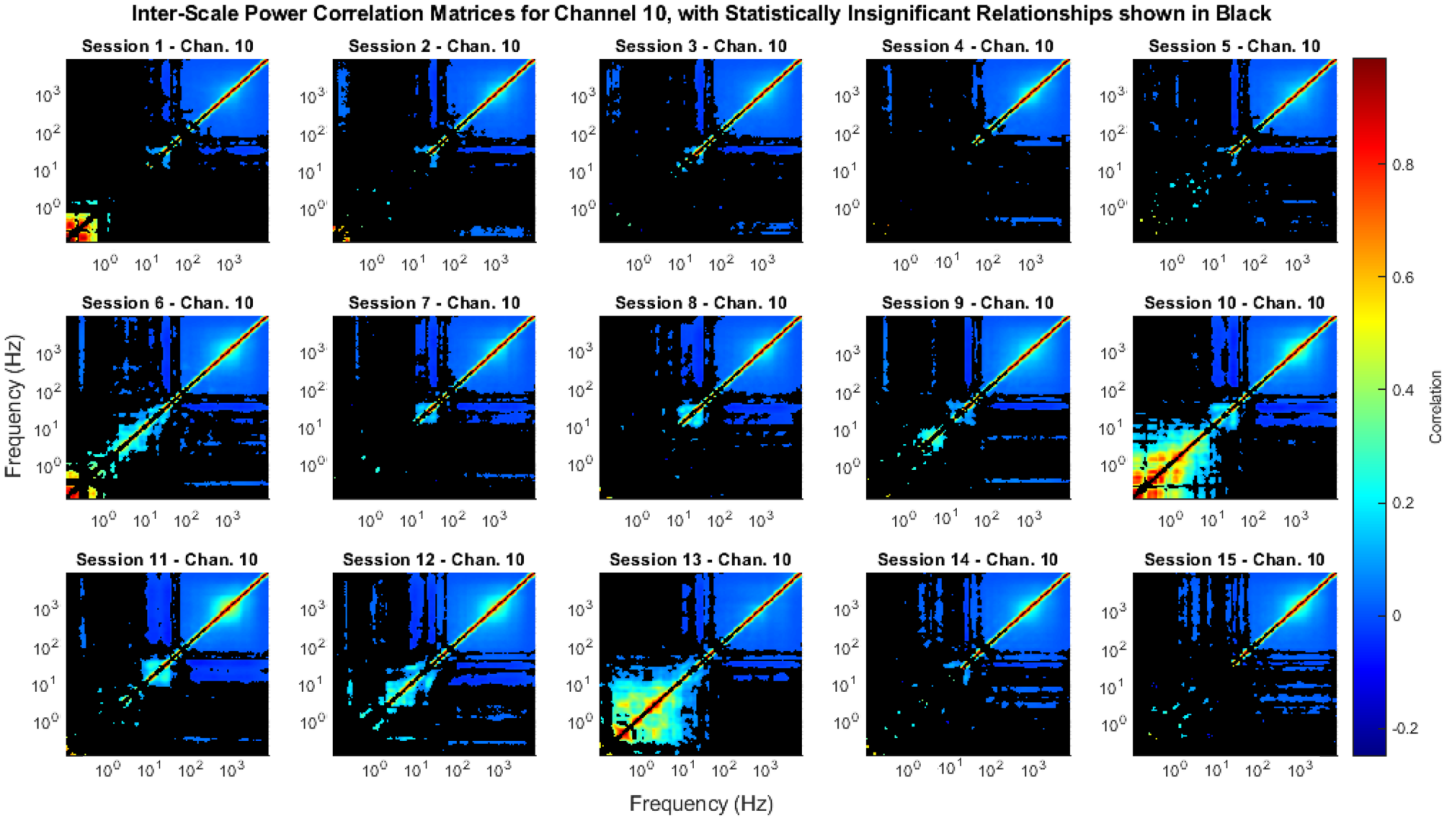
ISPCMs from Sessions 1–15 for Channel 10. These show the time-wise correlation between the power in various frequency bands, i.e. ISPCs. Non-statistically significant correlations, at an FDR $\alpha$ of 0.01, are shown in black.

**Figure 4:**
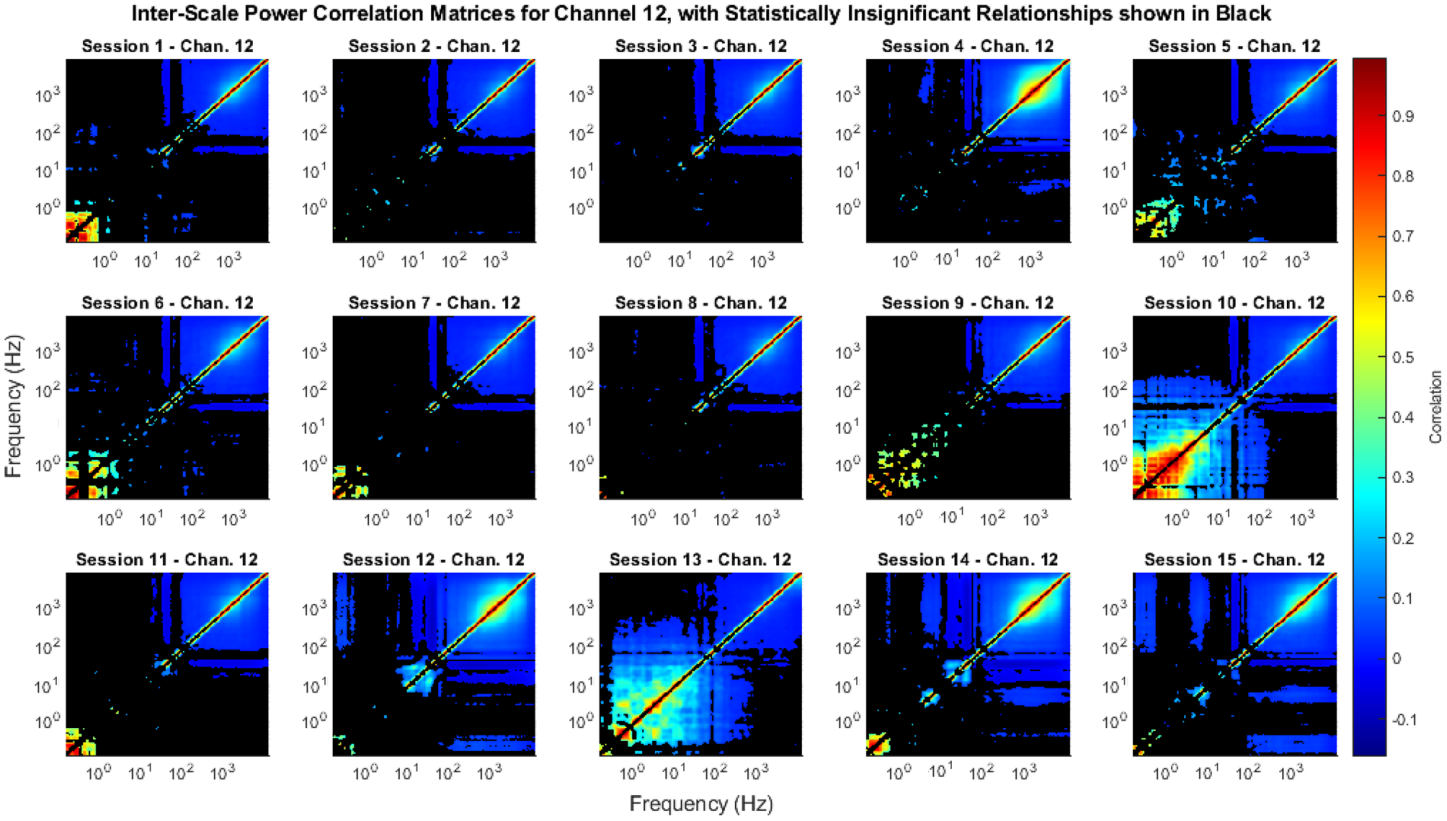
ISPCMs from Sessions 1–15 for Channel 12. Non-statistically significant correlations, at an FDR $\alpha$ of 0.01, are shown in black.

We calculated and tested the significance of the ISPCMs for 96 channels for 15 sessions each, for a total of 1440 broadband intracortical recordings (see “Methods” for dataset details). However, to avoid data-dredging any future work, we analysed only a subset of the results, i.e. channels 1–20, in this work. The complete set of ISPCMs *r* for all 96 channels and their associated figures are publicly available at^77^, and the associated code at both^77,78^. The full set of results analysed in this work include 300 ISPCMs. A subset of the statistically significant elements of the ISPCMs are shown in Figs. 3 and 4.

We define low frequencies as 0.1–10 Hz, medium frequencies as 10–100 Hz, and high frequencies as 100–7500 Hz. Individually for all recordings, we controlled the FDR at an $\alpha$ of 0.01.

### Neural processes reliably have signatures in frequencies as low as the beta band

**Figure 5:**
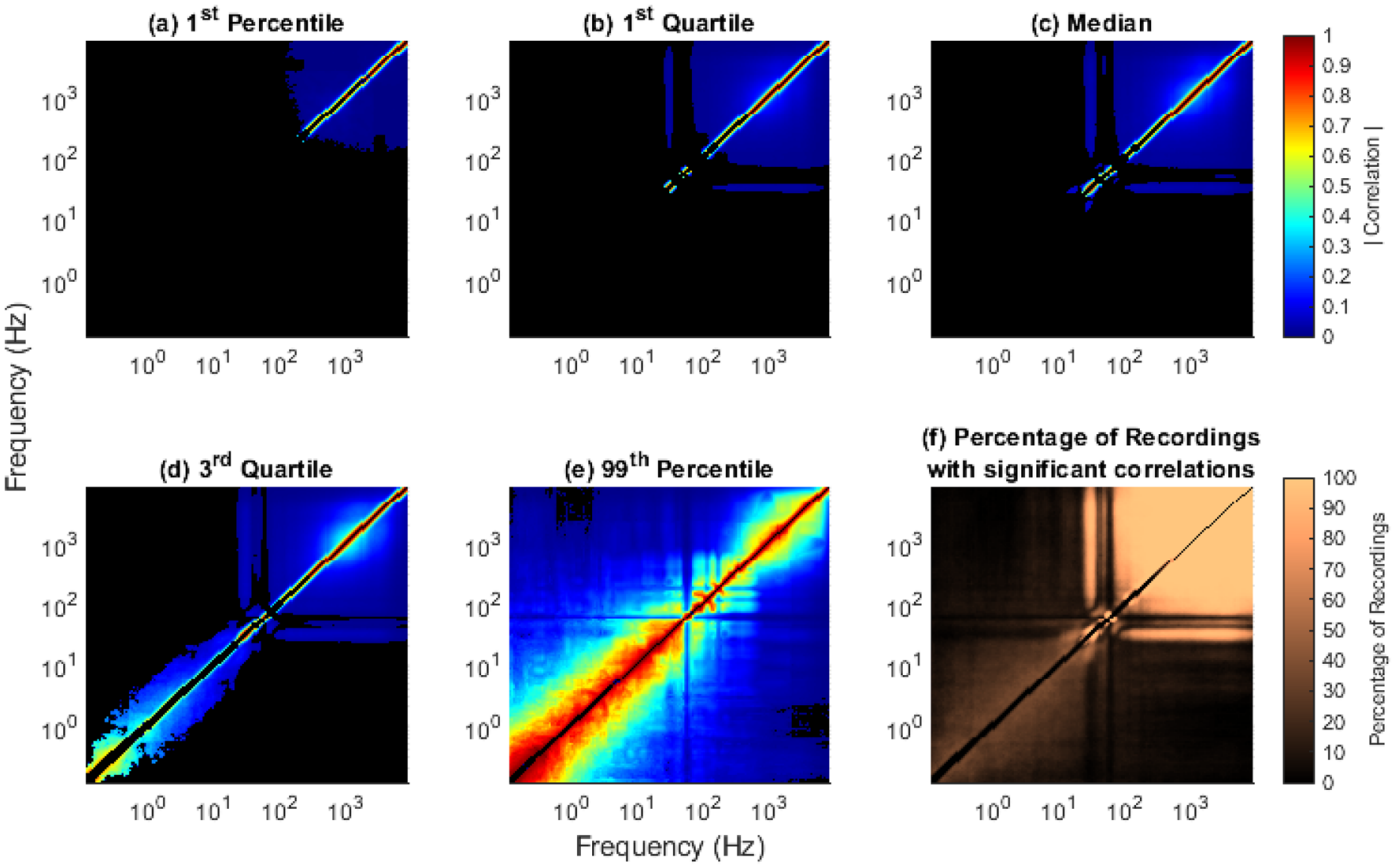
Meta-statistical inter-scale matrix plots, showing how many elements of the ISPCMs were significant across the analysed recordings. Black indicates non-significant ISPCs at an FDR $\alpha$ of 0.01. A total of 300 recordings were analysed, with 20 channels across 15 recordings. (**a**) 1st Percentile: for each ISPC, 99% of the analysed recordings had ISPCs above these values. The absolute values of the correlations were given here, as it is more intuitive in terms of relationship strength. (**b**) 1st Quartile: 75% of the analysed recordings had ISPCs above these values. (**c**) Median: 50% of the analysed recordings had ISPCs above these values. (**d**) 3rd Quartile: 25% of the analysed recordings had ISPCs above these values. (**e**) 99th Percentile: 1% of the analysed recordings had ISPCs above these values. The unique pattern is discussed in “Time-domain artifacts create strong low-mid frequency power correlations”. (**f**) Percentage of analysed recordings that had significant ISPCs.

The most interesting result is that high frequencies were found to be almost always correlated to high and medium frequencies. A significant correlation between signals suggests that they have a common input. As such, the results suggest that the power in these frequency bands have a common input, i.e. there are neural processes that have signatures in these frequency bands. In particular, even frequencies as low as 20 Hz, which correspond to the beta band, were found to be almost always correlated to kHz range frequencies. This supports findings in many other works that show that the power in various LFP bands is associated with spike activity^14–16,18–24,29,79^. In fact, the continuous range of the significant power correlations suggests that spikes have power signatures in a continuous range of frequencies from kHz down to 20 Hz. For individual recordings, this can be observed in Figs. 3 and 4. Across all of the analysed recordings, this can be observed in Fig. 5a–f. In particular, in Fig. 5b,f it can be seen that over 75% of analysed recordings had the same significant mid-high frequency power correlations, including the beta band.

The findings also support the concept of low-bandwidth encodings of neural information for BMI applications, as has been demonstrated by other works^2–4,7,8^. It warrants mentioning that the significant correlations are quite low. However, low inter-frequency power correlations are to be expected when sampling sparse neural processes at a high sampling resolution. This is because there may not be anything interesting happening in the vicinity of electrodes a large amount of the time. As a result, there is no common input to different frequency bands much of the time, which translates into low correlations overall. As such, the fact that the correlations are significant is generally more interesting than their magnitude. As aforementioned, it suggests that neural processes have signatures in those significantly correlated frequency bands.

It warrants mentioning that there is a noticeable lack of significant correlations to frequencies in the 55–65 Hz range (Fig. 5d,f). This is due to the pre-processing, which involved randomising the phases in that range (Supplementary Section 2). This was done to eliminate the effects of line noise.

### Few low-high frequency power correlations

To see how many low-high power correlations were significant, we compared the amount of significant low-high ISPCs to the total amount of tested low-high ISPCs for each recording. The graphical intuition is that we tested how many of the elements in the top left corner of the ISPCMs were significant for each recording. We found that there were relatively few low-high frequency power correlations in the results. In other words, the power in sub-10 Hz LFPs was generally not correlated to that in above-100 Hz frequencies.

**Figure 6:**
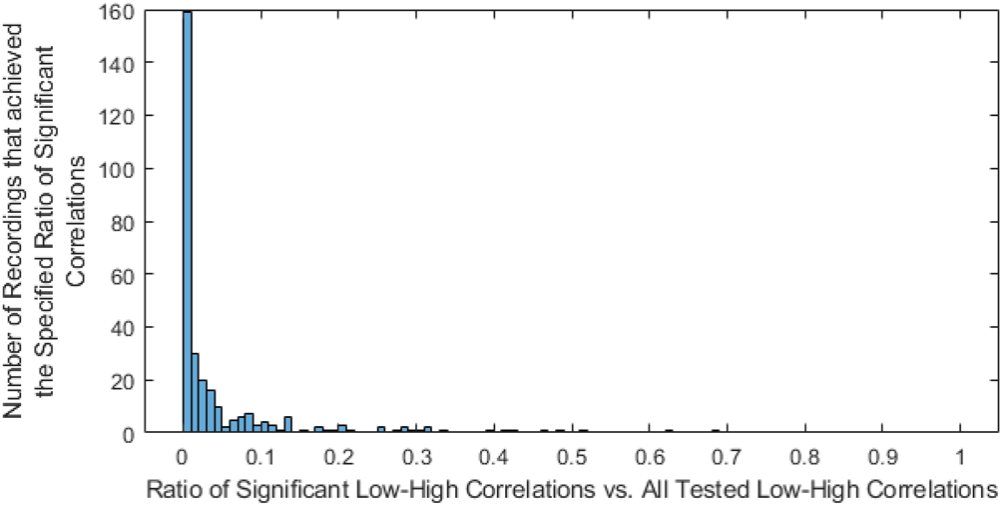
Histogram of the number of analysed recordings that achieved different ratios of significant-to-all-tested low-high correlations. The vast majority of recordings had almost no significant below-10 to above-100 Hz correlations. The FDR was controlled at an $\alpha$ of 0.01. A total of 300 recordings were analysed.

This is shown in Fig. 6, a histogram of the number of analysed recordings that achieved different ratios of significance relative to all tested low-high relationships. The vast majority of the analysed recordings achieved ratios that were near zero. This shows that significant low-high frequency power correlations are quite rare across the analysed recordings.

However, this is in direct disagreement with other studies. In Ref.^80^ they found that spike rates in Macaque M1 could be reliably estimated from sub-4 Hz LFP time-domain features, using robust statistical methods. There are at least two explanations for the difference between our M1 results and those of^80^. The first is that this work used the Fourier Transform method for the phase-randomisation. This may be overly conservative, as discussed further in Methods “Selection of phase randomisation method”. The second is that the datasets and feature extraction methods in question are different. This study used Utah arrays and Current Source Density (CSD) referencing, while^80^ used microwire electrodes and no referencing. As such, it warrants re-mentioning that different datasets and feature extraction methods will likely produce different results. This suggests that future work should involve comparing such different datasets and feature extraction methods.

### The vast majority of significant inter-scale power correlations were positive

**Figure 7:**
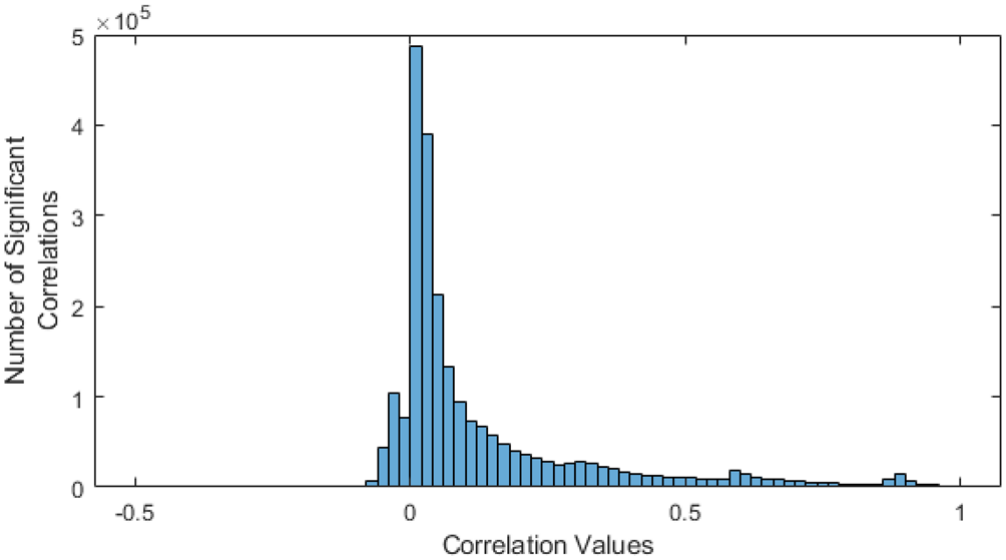
Histogram of significant correlation values across all analysed recordings. 88.4% of the significant correlations were positive.

Interestingly, the vast majority of significant ISPCs were positive (e.g. Figs. 3 and 4). I.e., when power went up in one frequency band, it generally went up in other correlated frequency bands, instead of down. This can be observed in Fig. 7, which shows that the vast majority of significant correlations (88.4%) were positive.

### Time-domain artifacts create strong low-mid frequency power correlations

**Figure 8:**
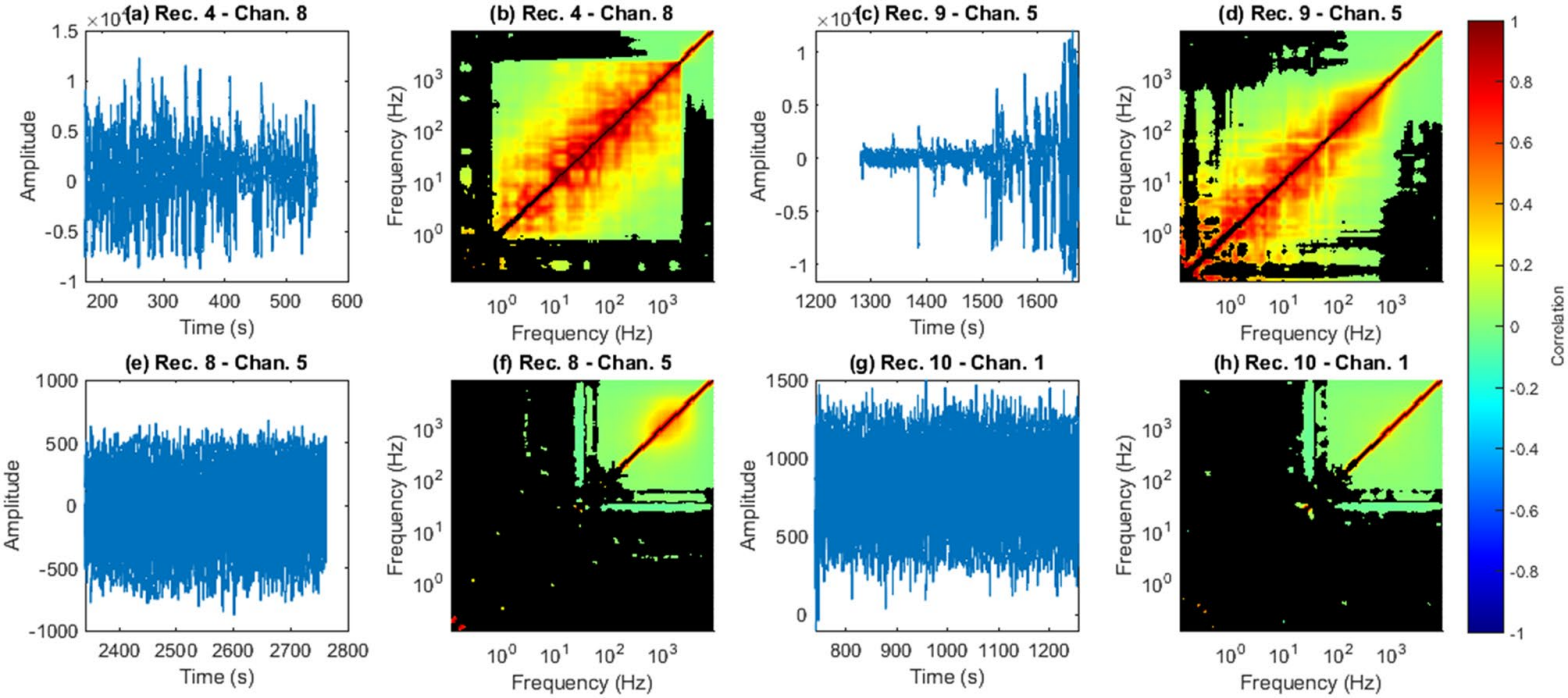
Time series and their associated inter-frequency maps from various session-channel pairs. In the inter-frequency maps, black indicates non-statistically significant relationships at an FDR $\alpha$ of 0.01. (**a**) and (**c**) contain strong time-series artifacts. Corresponding strong low-mid frequency power correlations are observable in (**b**) and (**d**). (**e**) and (**g**) contain no strong time-domain artifacts. (**f**) and (**h**) also contain no strong low-mid frequency power correlations.

Across all recordings, the comparison of time series and ISPC plots showed that signals with strong time-domain artifacts consistently had strong low-mid ISPCs (Fig. 8). Similarly, neural time series without strong artifacts consistently did not have strong low-mid ISPCs. There were 13 recordings that clearly had abnormally strong low-mid ISPCs, such as those shown in Fig. 8b,d. Of those 13, 10 were easily identifiable (e.g. Fig. 8a,c) based on observation of the time series in the author’s first attempt. As such, it seems likely that strong low-mid ISPCs result from recording artifacts and/or pathological activity.

### The neural signatures are chronically robust

These intra-channel relationships were generally quite robust across different recording sessions, i.e. across months. This is encouraging, as it suggests that the same neural processes are being recorded and are stable across long periods in this dataset.

## Discussion

### Significance of this work

There are only three possible sources of significant correlation in the power of different frequency bands in a neural signal. These are random chance, non-neural sources e.g. electronics noise, and neural sources. In this work, pre-processing is used to remove non-neural sources, in particular line noise. Then, a statistical significance test is used to determine how unlikely the observed result is to be due to random chance. As such, to the best of the author’s knowledge, this work gives us the first ever method to discover statistically significant ISPCs, due to neural sources, in broadband intracortical neural recordings.

This work is not intended to prove or disprove previous results in the neuroscience literature. This is because different datasets and different feature extraction methods may produce different results. The goal is to introduce a statistical test for inter-frequency power correlations that controls for some biases that are often not controlled for when inter-frequency relationships in non-stationary time-series are investigated, and to raise awareness of those biases. A secondary goal is to find statistically significant inter-frequency power correlations in the dataset in question.

As it relates to the secondary goal, the results of this work may still be contaminated by non-neural sources, potentially even biological sources. This can only be corrected by perfect pre-processing. This likely requires a strong familiarity with the neural recording set-up. As such, any inter-frequency analysis would benefit from an integrated understanding of how the data was gathered. Researchers are free to select from the publicly available results^77^ to determine what constitutes non-neural activity, based on their knowledge of the publicly available data^81^. In particular, dismissing the recordings with strong artifacts is likely a useful strategy. Plots of the Fourier transforms and time-domain signals, for each channel and session, are also provided at^77^.

The statistical significance test can also be used for other applications, e.g. biological signal processing, economics, finance, climatology, etc. It is appropriate for any application where one wishes to see if long-term trends are related to shorter-term components in a broad class of non-stationary time series, or what frequency bands contain covarying power.

### Possible improvements to the statistical test

Improvements to this test can likely be made. Accounting for lag is absent from the current numerical method, due to the computational expense. This is due to the massive amount of combinations of lag for different frequencies that are possible. Another obstacle is the difficulty of the multiple testing given multiple lag permutations. As such, all tested relationships in this work are instantaneous. In other words, the test does not capture ISPCs where there is a lag in the correlation between the two frequency bands in question.

While well-understood and computationally simple, the Pearson correlation coefficient is limited to finding linear, 1st order polynomial relationships. In this work, the Pearson correlation coefficient was used as the measure of association because its statistical significance characteristics are well understood. However, including other state-of-the-art association measures, capable of finding more general non-linear, non-functional relationships^82^, is an interesting avenue for future research. The measure of association must however be normal under the null for the method in^76^ to be appropriate.

As discussed in “Methods”, a clipping method is used to mitigate the effects of cyclic discontinuities during phase-randomisation. This makes the method incompatible with strongly monotonous signals. Classical windowing instead of clipping could broaden the class of non-stationary signals that the method could be applied to. However, this requires further investigation to understand the contribution of windowing on the ISPCM. This is discussed further in “Methods”.

Investigating the use of other TFDs would also likely be useful.

### Do only correlated frequency bands contain interesting information?

With sufficiently good pre-processing, only neural sources and random chance, controlled at some level, contribute to the significant elements of the ISPCM. Here we will argue that if the neural process is not represented in the significant elements of the ISPCM, it may be that it is not statistically measurable.

Assume $\mathbf {x}$ is a neural recording measuring biological, presumably neural, processes. In the TFD of $\mathbf {x}$, assuming good enough time-frequency resolution and that the recorded processes have a consistent signature distinguishable from noise, we should see roughly the same signatures in the same frequencies whenever the same biological process occurs under the same conditions. We also assume that biological processes have signatures in multiple/broad frequency bands. The alternative would be a pure sinusoid electronic signature, which seems biologically implausible. To be clear, the frequency bands can be adjacent to each other in the TFD. The term frequency bands here does not signify historically discovered neural frequency bands, e.g. alpha, beta, delta, gamma, etc. It signifies scales in the TFD, i.e. columns in the WPS matrix.

In a long enough recording, if 2 otherwise random signals are synchronized often enough, they will become statistically significantly correlated relative to noise with no synchronization. As such, we can say that in a long enough recording $\mathbf {x}$, if there are biological processes (1) whose signatures are distinguishable from noise, and (2) which occur often enough, then in a TFD of $\mathbf {x}$, the frequency bands that contain the signatures of the processes will become statistically correlated relative to random chance.

The author’s hypothesis is that only the statistically correlated frequency bands contain interesting neural information. This is because virtually all measurable biological processes presumably produce, in long enough recordings, statistical correlations between the frequency bands of their signatures. If there are no significant inter-frequency power correlations for a given frequency band, then there is no biological process indistinguishable from noise that has that frequency band as a signature. This is because that process would likely also have other frequency bands as its signatures, which would produce a correlation between the signature frequencies. Therefore, looking at the statistically significant inter-frequency power correlations should allow us to identify the subset of frequency bands that contain biological information. It may even allow us to rank the frequency bands by informativeness without having to do any decoding to an external metric, e.g. behavior. To the best of the author’s knowledge, this has never been achieved before. Caveats to this claim are lag and non-linearity in the inter-frequency relationship as discussed in “Possible improvements to the statistical test”.

The key highlight is that we hypothesize that if a frequency band does not have a significant power correlation to another frequency band, that indicates that it is likely not a signature of any neural process measured in the signal. As such, this test may enable us to discover every interesting frequency band in the neural signal.

### Future work

Future work will include a Feature of Importance analysis to test the hypothesis in “Do only correlated frequency bands contain interesting information?”. We will test whether the frequency bands with few statistically significant ISPCs have significantly worse behavioral decoding performance than those with more. This will allow us to answer whether significant ISPCs, along with pre-processing, are a reliable method for extracting the frequency-signatures of neural processes. If successful, this may open the door to a state-of-the-art encoding of neural information based on ISPCs. It may also allow us to discover every interesting frequency band in a neural signal.

## Methods

The bulk of this work was done using MATLAB R2020b. Most of the computations were done on the Imperial College London High Performance Computing cluster. All results and code from this work have been made publicly available at Zenodo^77^, and the a copy of the code is at Github^78^. The code can be easily applied to any arbitrary signal, given the conditions in “Signal conditions for the use of this test” are met.

### Dataset

#### Dataset general characteristics

In this work, we analysed the publicly available 2016–2017 Sabes Lab Broadband recordings described in detail at^81^. To summarise, this dataset contains long-term, continuous recordings from a 96-channel silicon microelectrode ‘Utah’ array (Blackrock Microsystems, Salt Lake City, UT). These were implanted in the Primary Motor Cortex M1. The subject ‘Indy’ was an adult male Rhesus macaque monkey, performing a free reaching task.

The recordings are across 30 sessions. They are not segmented into trials, and have an average duration of $\sim$ 520 s. As such, they enable us to reliably analyse low-frequency aspects of the neural signals. In this work, we analysed the first 15 sessions in chronological order, from 2016-06-24-03 to 2016-10-17-02. These had an average length of $\sim$ 480 s. Only channels 1 to 20 were analysed, so that future work on these sessions is not data dredged.

#### Dataset frequency characteristics

In this dataset, the broadband data was pre-amplified with a PZ2 Preamplifier (Tucker-Davis Technologies, Alachua, FL) with a frequency response of 3 dB: 0.35 Hz–7.5 kHz; 6 dB: 0.2 Hz–8.5 kHz^83^. There was also an anti-aliasing filter built-in to the pre-amplifier: 4th order low-pass with a roll-off of 24 dB per octave at 7.5 kHz. It was then sampled at $F_s = 24414.0625$ Hz and 16-bit resolution using a RZ2 BioAmp Processor (Tucker-Davis Technologies, Alachua, FL). There is an anti-aliasing filter built-in to the recording amplifier: 2nd order low-pass with a roll-off of 12 dB per octave at 7.5 kHz. As such, the recording configuration allows us to observe inter-frequency power correlations between $\sim$ 0.2–7500 Hz.

### Data pre-processing

#### Spectral interpolation and phase randomisation for line noise removal

There are three main sources of noise in neural recordings: electrode impedance thermal noise, tissue thermal noise and interface electronics noise^84^. Of the three, only interface electronics noise is likely to contain correlated power across different frequency bands. This is the aspect of noise that will impact our results the most. A clear example of interface electronics noise that produces correlated power in different frequency bands is line noise and its harmonics.

In this work, we used an adjusted Spectral Interpolation (SI) method to remove the line noise^85^. This avoided the distortion associated with commonly used notch filters, e.g. Gibbs ringing. Since we are particularly concerned with inter-frequency power correlations, we differed from^85^ in that we also phase-randomised the interpolated frequencies. Using the SI method, we removed each line noise harmonic up to the Nyquist frequency, as well as a few other noticeable components. The technical details are given in the Supplementary information (Supplementary Section 2).

#### Current source density referencing of electrodes

Neural recordings can contain contributions from distant neural activity^29,86^. Therefore, to bias the recording towards local processes, the spectrally interpolated signals were referenced using CSD referencing, i.e. Laplacian referencing^87,88^. The technical details of the CSD procedure are given in the Supplementary Information (Supplementary Section 3).

#### Standardizing the length of the recordings

The statistical significance test requires that many white noise processes are produced. These must be close to the same length as the analysed signal. To reduce the total amount of MC simulations in this work, the CSD-referenced neural recordings were shortened to a standard length. If the signal was between 350 and 400 s in length, it was shortened to 350 s. If it was between 400 and 500 s in length, it was shortened to 400 s. If it was above 500 s, it was shortened to 500 s. This was done by removing samples from the end of *x* so that $n = F_s \times 350$, $n = F_s \times 400$ or $n = F_s \times \; 500$.

#### Clipping of data

The end of the neural data *x* was clipped so that the last kept sample of *x* was within 1% of $x_1$. It was then zero-meaned. This was done so as to somewhat lessen the effects of cyclic discontinuity edge effects during phase-randomisation of the scales, although it is not obvious this is necessary for our application. The technical details are given in the Supplementary Information (Supplementary Section 4).

### Data post-processing

#### Continuous wavelet decomposition and the morse wavelet

Let $\mathbf {x} \; \{x_k \; \epsilon \; {\mathbb {R}} \; | \; k \; \epsilon \; \mathbb {N^+} \; | \; 1 \le k \le n\}$ be a discrete time series with equal time spacing $\Delta t$, where *n* is the number of samples. For the CWT on a discrete sequence^60,65^, the decomposition coefficients $W(a,\tau )$ are obtained by decomposing $\mathbf {x}$ over scaled and translated version of the mother wavelet function $\psi$:

2 $$\begin{aligned} W(a,\tau ) = \frac{1}{\sqrt{a}} \sum \limits ^n_{k=1} x_k \; \psi ^{*}\left( \frac{(k-\tau )\Delta t}{a}\right) , \end{aligned}$$

where $a \; \{a \; \epsilon \; \mathbb {N^+} | \; 1 \le a \le u \}$ denotes the scale, and *u* is the number of scales in the CWT decomposition. $\tau \; \{\tau \; \epsilon \; \mathbb {N^+} | \; 1 \le \tau \le n \}$ denotes the translation. * denotes complex conjugation.

For our CWT wavelet, we selected the continuous and analytic Morse wavelet at $\gamma$ = 3, known as the Airy wavelet. It has a number of desirable properties, e.g. almost minimal Heisenberg area, unique mapping of scale to frequency, etc.^73^. However, when looking at covarying power between frequency bands in terms of the power, we are uniquely interested in wavelet power spectra statistics. As such, the results will be similar across wavelets. This is because the choice of wavelet is generally not critical for wavelet power spectra analysis^68^.

The decomposition coefficients for the Morse wavelet^89^ are given by:

3 $$\begin{aligned} W_{B,\gamma }(a,\tau ) = \frac{1}{\sqrt{a}} \sum \limits ^n_{k=1} x_k \; \psi _{B,\gamma }^{*}\left( \frac{(k-\tau )\Delta t}{a}\right) , \end{aligned}$$

where the order *B* controls the low-frequency behaviour, and the family $\gamma$ controls the high-frequency decay.

In this work, we used the default MATLAB value of $B = 20$. For more details on the Morse wavelet, see^90,91^. Being an analytic wavelet, it produces complex coefficients. To get the WPS coefficients **S**, one takes the element-wise square of the absolute value of the complex wavelet coefficients^68^:

4 $$\begin{aligned} {\mathbf {S}} = (s_{a,k})_{u\times n} = |W_{B,\gamma }|^2. \end{aligned}$$

This gives one the estimated power at each scale and moment in time. In this work, scale is taken to be a frequency band, or the time-wise vector of WPS coefficients at the given scale index. This is because of the unique mapping of scale to frequency band by the Morse wavelet^73^. Frequency is taken to be a frequency, many of which can be sampled in a scale.

#### COI

The CWT has a feature called the Cone of Influence (COI). This refers to the zone of the WPS where edge effects are not present and the coefficients can be relied upon to be accurate. Outside the COI, the stretched wavelet contains the contributions of padded samples outside $\mathbf {x}$. Wider scales use more time-samples in their calculation than narrower scales. Therefore, the zone in which wider scales extend beyond $\mathbf {x}$ is larger than for narrower scales. A visual example of the COI is shown in Fig. 1b.

#### Pearson correlation coefficient

Let $\mathbf {s_{a}}$ denote the vector containing the coefficients of matrix $\mathbf{S }$ from scale index *a*, $\mathbf {s_{a}} = s_{a,1}, \ldots \, ,s_{a,k}$. The inter-scale Pearson correlation $r_{a,b}$, i.e. ISPC, between two WPS scales $\mathbf {s_a}$ and $\mathbf {s_b}$ is given by:

5 $$\begin{aligned} r_{a,b} = \frac{\sum \limits _{k=1}^n \left( s_{a,k} - \bar{s_a}\right) \left( s_{b,k} - \bar{s_b}\right) }{\sqrt{\sum \limits _{k=1}^n \left( s_{a,k} - \bar{s_a}\right) ^2} \sqrt{ \sum \limits _{k=1}^n \left( s_{b,k} - \bar{s_b}\right) ^2}}, \end{aligned}$$

where $\bar{s_{a}} = \frac{1}{n} \sum \limits _{k=1}^n s_{a,k}$, and $\{a,b \; \epsilon \; \mathbb {N^+} | \; 1 \le a,b \le u \}$. **R** denotes the full matrix of ISPCs, i.e. the ISPCM, with $\mathbf{R } = (r_{a,b})_{u \times u}$.

Only samples within the COI are reliable. As such, WPS scales for which less than 90% of the samples were within the COI were removed. Then, samples were removed from the beginning and end of the WPS so that, at any remaining scale, no sample was within the COI. As such, each ISPC was calculated using only samples that were within the COI for both scales. This method was thousands of times more computationally effective than taking the pairwise correlation between all samples within the COI. For all recordings, the remaining scales included 0.11 to 8500 Hz.

#### Data post-processing summary

**Figure 9:**
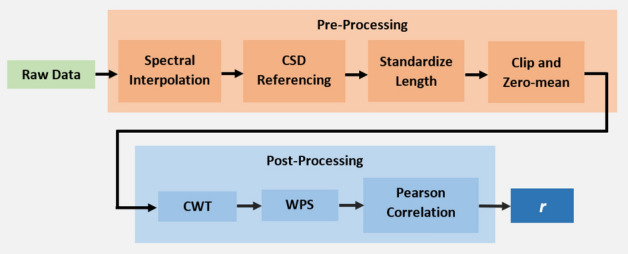
Diagram of pre- and post-processing data flow for the neural data.

The data post-processing involved CWT-decomposing the pre-processed data with Eq. (3), extracting the Wavelet Power Spectrum coefficients **S** with Eq. (4), and finally calculating the ISPCM **R** via Eq. (5) and as described in “Pearson Correlation Coefficient”. The complete data flow for pre- and post-processing of the neural recordings is given in Fig. 9. An example of a neural data ISPCM and associated WPS is shown in Fig. 1a,b.

### CWT inter-scale power correlation significance test implementation

#### Estimating the mean white noise inter-scale power correlation matrix

**Figure 10:**
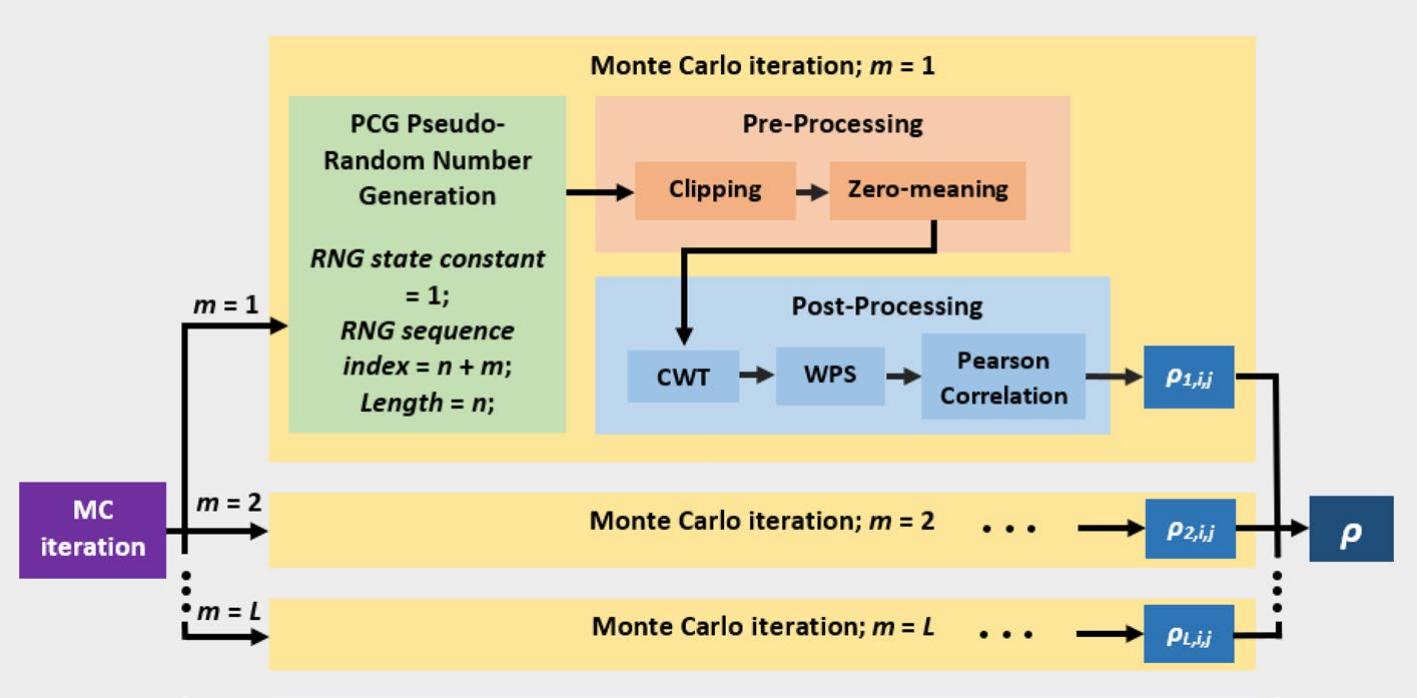
Diagram of MC data flow for determining white noise CWT-contribution inter-scale power correlation matrix.

To generate the first null element in our test statistic, i.e the mean white noise ISPCM, we performed a MC simulation. This produced *L* = 1000 of random white noise signals for each standard recording length (i.e. 350, 400 and 500 s). The generation of the white noise signals is discussed in the Supplementary Information (Supplementary Section 5). For each MC white noise signal, we followed the same post-processing steps as for the neural CSD-referenced and shortened data. We performed the CWT, and calculated the random white-noise WPS coefficients ${\mathbf {Q}}$ = $(q_{v,a,k})_{L \times u\times n}$. Let $\mathbf {q_{v,a}}$ denote the vector containing the coefficients of matrix $\mathbf{Q }$ from scale index *a* and MC iteration *v*, $\mathbf {q_{v,a}} = q_{v,a,1}, \ldots \,,q_{v,a,k}$. The inter-scale Pearson correlation $\rho _{v,a,b}$ between two scales $\mathbf {q_{v,a}}$ and $\mathbf {q_{v,b}}$, from MC iteration $v \, \{v \; \epsilon \; \mathbb {N^+} | \; 1 \le v \le L \}$, is given by:

6 $$\begin{aligned} \rho _{v,a,b} = \frac{\sum \limits _{k=1}^n \left( q_{v,a,k} - \bar{q_{v,a}}\right) \left( q_{v,b,k} - \bar{q_{v,b}}\right) }{\sqrt{\sum \limits _{k=1}^n \left( q_{v,a,k} - \bar{q_{v,a}}\right) ^2} \sqrt{ \sum \limits _{k=1}^n \left( q_{v,b,k} - \bar{q_{v,b}}\right) ^2}}, \end{aligned}$$

where $\bar{q_{v,a}} = \frac{1}{n} \sum \limits _{k=1}^n (q_{v,a,k})$ denotes the mean of $\mathbf {q_{v,a}}$. *n* depends on whether the MC process is 350, 400 or 500 s long, and on the clipping in MC iteration *v*. The combined data flow for the CWT-contribution MC is shown in Fig. 10. The mean ISPCM was then taken for each standardised recording length: $\bar{\rho _{a,b}} = \sum \limits _{v=1}^L \rho _{v,a,b}/L$. As an example, Fig. 12a,b (“Conservatively removing the effects of the frequency-domain oversampling”) gives the mean MC-derived ISPCM for white noise processes of length 500 s.

#### Generating the normal distribution due to intra-scale autocorrelation

##### Selection of phase randomisation method

Phase-randomisation is used to create surrogate signals that have the same statistical properties as the original signal, but are otherwise random. It is commonly performed, as it is in this work, to determine the significance of the cross-correlation between autocorrelated signals^8,41,43–46^. There are multiple methods for phase-randomization. The two most common methods, for stationary signals, are the Fourier Transform (FT) method and the more advanced Iterated Amplitude Adjusted Fourier Transform (IAAFT) method^92^.

For non-stationary signals, a phase-randomisation method was proposed in^93^. It randomises the phases of the CWT of the signal while preserving the magnitudes of the coefficients, and then takes the ICWT. This largely preserves the time-frequency structure of the signal. As a result, any non-stationary structure is largely respected in the surrogate signals. However, when testing inter-frequency power correlations, it is not obvious that one wishes to maintain any non-stationary structure within the power of each frequency band. If the non-stationarity varies significantly similarly between 2 separate frequency bands, that is interesting. It is the sort of relationship one is looking for, and so controlling for it in the surrogate data seems counter-intuitive. As such, only the FT and IAAFT methods were considered.

**Figure 11:**
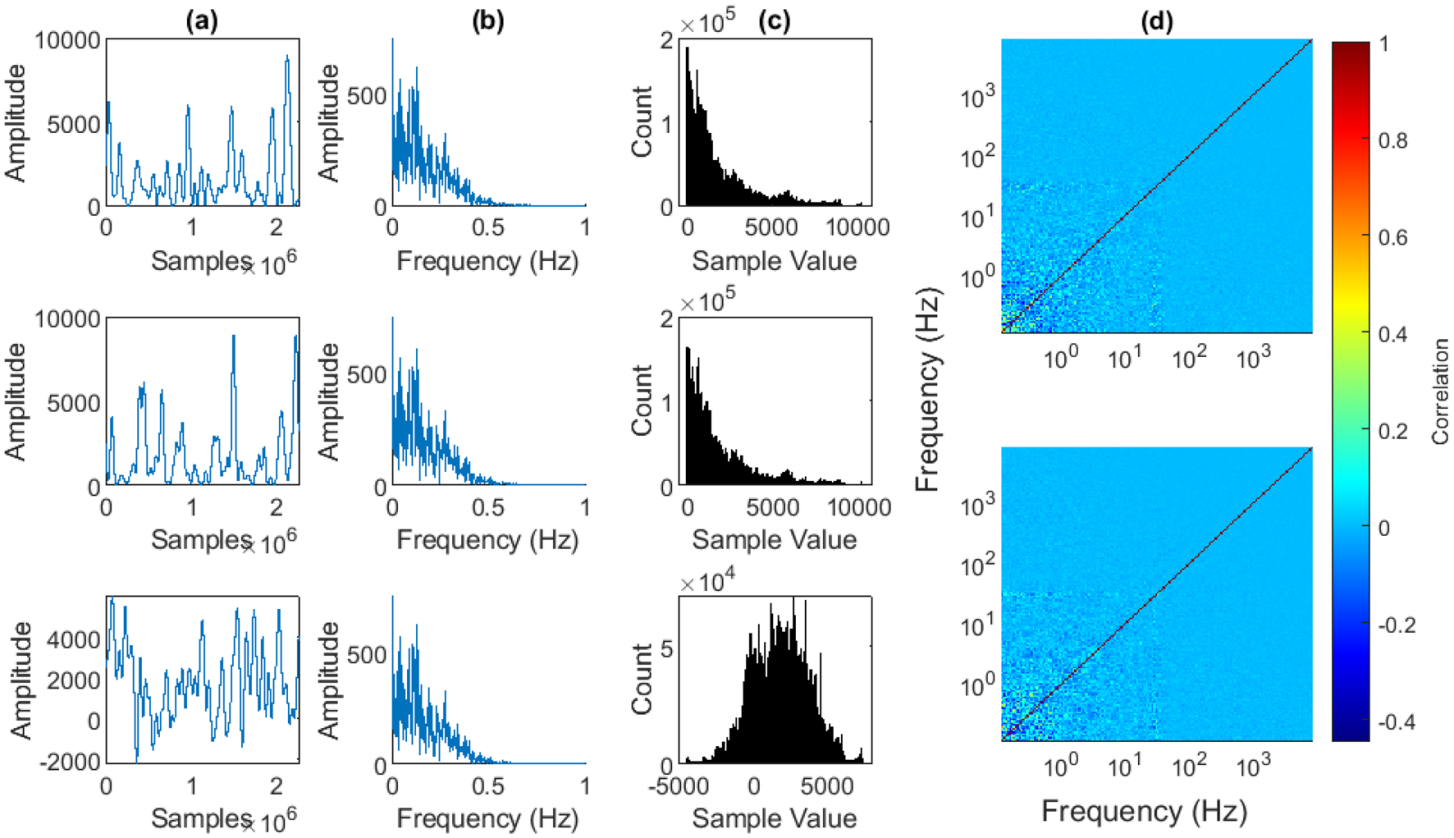
Effects of IAAFT and FT phase randomisation on intra-scale data. Top: original neural data from Session 1, Chan. 1, Scale 130 (central frequency of 1.1 Hz). Middle: IAAFT phase-randomised data. Bottom: FT phase-randomised data. (**a**) Time-series sample. (**b**) Fourier transform. (**c**) Time-series histogram. (**d**) Phase-randomised ISPCM example for IAAFT (top) and FT method (bottom), from Session 1, Chan. 1.

The FT method involves taking the Fourier transform of the signal, randomising the phases, and transforming the data back via the IFFT. This preserves the first order autocorrelation properties of the signal. However, unlike the IAAFT, it does not maintain the sample histogram. As such, phase-randomised surrogates often took negative values, even though original $\mathbf {s_a}$ values are strictly non-negative. Figure 11 shows examples of the original, IAAFT and FT phase-randomised time series, Fourier transform and sample histograms, and the phase-randomised ISPCM for both methods. It can be observed that the Fourier transform and histogram are maintained by the IAAFT phase-randomisation. However, only the Fourier transform is maintained by the FT method.

Ultimately, the choice of the FT method over the IAAFT was due to the normality of the test statistics. The multiple testing method proposed in Ref.^76^ requires normal distributions under the null, and the inter-scale distributions produced by the IAAFT method were found to often be non-normal (Figures not shown, but the IAAFT null distributions are available for download at^77^). Observation (Supplementary Fig. 1a–c), followed by normality testing (Supplementary Fig. 1d–i), of the histograms of the FT nulls revealed that the vast, vast majority were normally distributed at an FDR $\alpha$ of 0.05. As such, they were compatible with the multiple testing procedure from^76^.

As such, the sample histograms were sacrificed for the sake of normality. Therefore the presented results are from the FT method. However, the final results of the statistical testing were compared for the two methods. The FT method found slightly fewer significant results, making it more conservative. In particular, the IAAFT method often found more low-high frequency power correlations to be significant than the FT method. As a result, there is some ambiguity in the results, particularly as it concerns low-high frequency power correlations.

##### Producing the null distributions from the FT method

As such, to generate the second null distribution, for each recording WPS **S**, for each scale $\mathbf {s_a}$, the phases were FT randomised for each MC iteration $h \, \{h \; \epsilon \; \mathbb {N^+} | \; 1 \le h \le H \},\, H \approx 250$. This was a sufficient number of iterations to reliably determining the mean and standard deviation of the normal distribution. We denote the phase randomised version of $\mathbf {s_a}$ in MC iteration *h* as $\mathbf {\mu _{h,a}}$. The inter-scale Pearson correlation $\phi _{h,a,b}$ between two scales $\mathbf {\mu _{h,a}}$ and $\mathbf {\mu _{h,b}}$ is given by:

7 $$\begin{aligned} \phi _{h,a,b} = \frac{\sum \limits _{k=1}^n \left( \mu _{h,a,k} - \bar{\mu _{h,a}}\right) \left( \mu _{h,b,k} - \bar{\mu _{h,b}}\right) }{\sqrt{\sum \limits _{k=1}^n \left( \mu _{h,a,k} - \bar{\mu _{h,a}}\right) ^2} \sqrt{ \sum \limits _{k=1}^n \left( \mu _{h,b,k} - \bar{\mu _{h,b}}\right) ^2}}, \end{aligned}$$

where $\bar{\mu _{h,a}}$ is the mean of $\mathbf {\mu _{h,a}}$. There are *H* elements in $\mathbf {\phi _{a,b}}$ for each (*a*, *b*), where $\mathbf {\phi _{a,b}} = \phi _{1,a,b}, \ldots \, , \phi _{H,a,b}$.

#### Summing the elements to produce the null distribution

For each recording, the two elements were added together to form the final null distribution, $\mathbf {\chi _{a,b}}$:

8 $$\begin{aligned} \mathbf {\chi _{a,b}} \sim N \bigg ( \bar{\rho _{a,b}}+\bar{\phi _{a,b}}, Var(\mathbf {\phi _{a,b}})\bigg ), \end{aligned}$$

where $Var(\mathbf {\phi _{a,b}})$ represents the sample variance of $\mathbf {\phi _{a,b}}$, $\bar{\phi _{a,b}} = \frac{1}{H}\sum \limits ^{H}_{h} \phi _{h,a,b}$, and $\bar{\rho _{a,b}} = \frac{1}{L}\sum \limits ^{L}_{v} \rho _{v,a,b}$. The $\rho$ distribution in question depends on the standardised length of the analysed recording. This is because in practice there were 3 different versions of $\rho _{a,b}$: one for each standard MC signal length (350, 400, 500 s).

The test statistic $T_{a,b}$ is then obtained by standardizing $r_{a,b}$ by the mean and standard deviation of the final normal null distribution $\mathbf {\chi _{a,b}}$,

9 $$\begin{aligned} T_{a,b} = \frac{r_{a,b} - \bar{\chi _{a,b}}}{\sqrt{Var(\mathbf {\chi _{a,b}})}}. \end{aligned}$$

#### Multiple testing of test statistics under dependency

The second part of the method in Ref.^76^ involves performing multiple testing on the asymptotically normal test statistics. We test which elements of $T_{a,b}$ are significantly different from the standard normal distribution. The procedure is as follows^76^:

1. Calculate the test statistics $T_{a,b}$ for $1 \le a < b \le u$, in our work via the MC method above.
2. Obtain the threshold ${\hat{t}}$, above which we reject the hypotheses. To do so, first we calculate how many hypotheses *T* are rejected, *R*(*t*), for threshold value *t*, $0 \le t \le d_u$, where $d_u = \sqrt{4 \log u - 2 \log ( \log u)}$.
  10 $$\begin{aligned} R(t) = \sum \limits _{1 \le a < b \le u} I\left\{ |T_{a,b}| \ge t \right\} , \end{aligned}$$
  where $I\left\{ \cdot \right\}$ denotes the indicator function. Second, we calculate the threshold ${\hat{t}}$.
  11 $$\begin{aligned} {\hat{t}} = \inf \left\{ 0 \le t \le d_u \, : \frac{G(t)(u^2 - u)/2}{R(t) \vee 1} \le \alpha \right\} , \end{aligned}$$
  where inf denotes the infimum, and $G(t) = 2- 2\Phi (t)$, where $\Phi (t)$ is the cumulative distribution function of the standard normal distribution. If Eq. (11) does not produce a value for ${\hat{t}}$, set ${\hat{t}} = 2\sqrt{\log u}$.
3. Reject $$H_{0,a,b}$$ whenever $$|T_{a,b}| \ge {\hat{t}}$$.

As such, we reject all $H_{o,a,b}$ with associated $|T_{a,b}| \ge {\hat{t}}$. This indicates that, when the FDR is controlled at $\alpha$ under dependency, the rejected inter-scale relationships are statistically significant.

### Signal conditions for the use of this test

For a signal to be compatible with the proposed test for inter-frequency power correlations, there are some conditions that need to be met. Firstly, the Fourier Transform of the signal must exist. To be more specific, the following Dirichlet conditions are sufficient but not necessary^94^:

- The signal must have a finite number of maxima and minima over any finite interval.
- The signal should have a finite number of discontinuities over any finite interval.
- The signal should be absolutely integrable.

There are signals that violate one or more of the Dirichlet conditions and for which the Fourier Transform exists, e.g. the unit step. As such, the above conditions are considered sufficient, but not necessary. However, if they are met then the signal has a Fourier Transform, and by extension a TFD can be applied. A general condition is that if the signal can be produced in laboratory, i.e. it has finite energy, its Fourier Transform exists^94^.

Additionally, the phase-randomisation procedure must be applicable within each frequency band concerned. Since the phase-randomisation method requires the use of a Fourier Transform, the same Dirichlet conditions apply to each frequency band.

Secondly, the multiplicity testing conditions from^76^ must be met: the nulls tested against must be normal, and the dependency between test statistics should not be too large. If the generated nulls via the phase-randomisation method are non-normal, then the testing procedure in the proposed method is not appropriate. If the test statistics are highly dependent, then the test lacks some power. However, this is quite a weak condition^76^.

Thirdly, the method uses a clipping method to somewhat mitigate the effects of cyclic discontinuities during phase-randomisation (discussed in Supplementary Section 4). This assumes that there is a segment of sufficient length in the signal *x* between indices $k_1$ and $k_2$, where $x_{k_1} \approx x_{k_2}$ and $1 \le k_1 \ll k_2 \le n$, that can be and is selected during clipping. If there is no such segment that can be used, then one could use some classical windowing function that does not introduce too much non-linear distortion in the correlation coefficient normalisation and the CWT. In “Validation of the test on synthetic data” it was found that the Hanning window is a good candidate.

If a windowing function is used, the results should be interpreted with this in mind. In particular, one should be aware that the correlation coefficient will be distorted because the standard deviation of the windowed signal is smaller at the edges of the window than in the middle. As such, windowing disproportionately weights contributions to the inter-frequency power correlation from the middle of the signal. Where possible, the clipping method should be used as it causes less distortion in the correlation coefficient (see Supplementary Fig. 4).

As such, for the proposed method to be used, the following conditions need to be met. Firstly, it is sufficient but not necessary that the Dirichlet conditions apply to both the original signal and to the time-varying power signal within each frequency band considered. Secondly, the criteria in Ref.^76^ must also be met. Thirdly, the clipping method is inappropriate if there is no segment of sufficient length in the signal between some indices $k_1$ and $k_2$, where $1 \le k_1 \ll k_2 \le n$ and $x_{k_1} \approx x_{k_2}$. If so, then a classical windowing function should perhaps be used and the results interpreted with this is in mind. The author cannot think of another category of non-stationary signal to which the proposed method would not apply.

### Conservatively removing the effects of the frequency-domain oversampling

In this work, we added the white noise mean ISPCM to the distribution of ISPCMs derived from phase-randomised versions of **S**. This is equivalent to deleting the white noise mean ISPCM from the measured neural ISPCMs. This is a very conservative method to account for the bias of the frequency domain oversampling. For example, if a neural ISPC equals 1, and the mean value for white noise input is 0.8, the adjusted value will be 0.2. This value may not be significant in comparison to the phase-randomised null distribution, whose values can in theory range between − 1 and 1, and is centered at 0. As such, even a measured perfect correlation may not be significant in our test.

**Figure 12:**
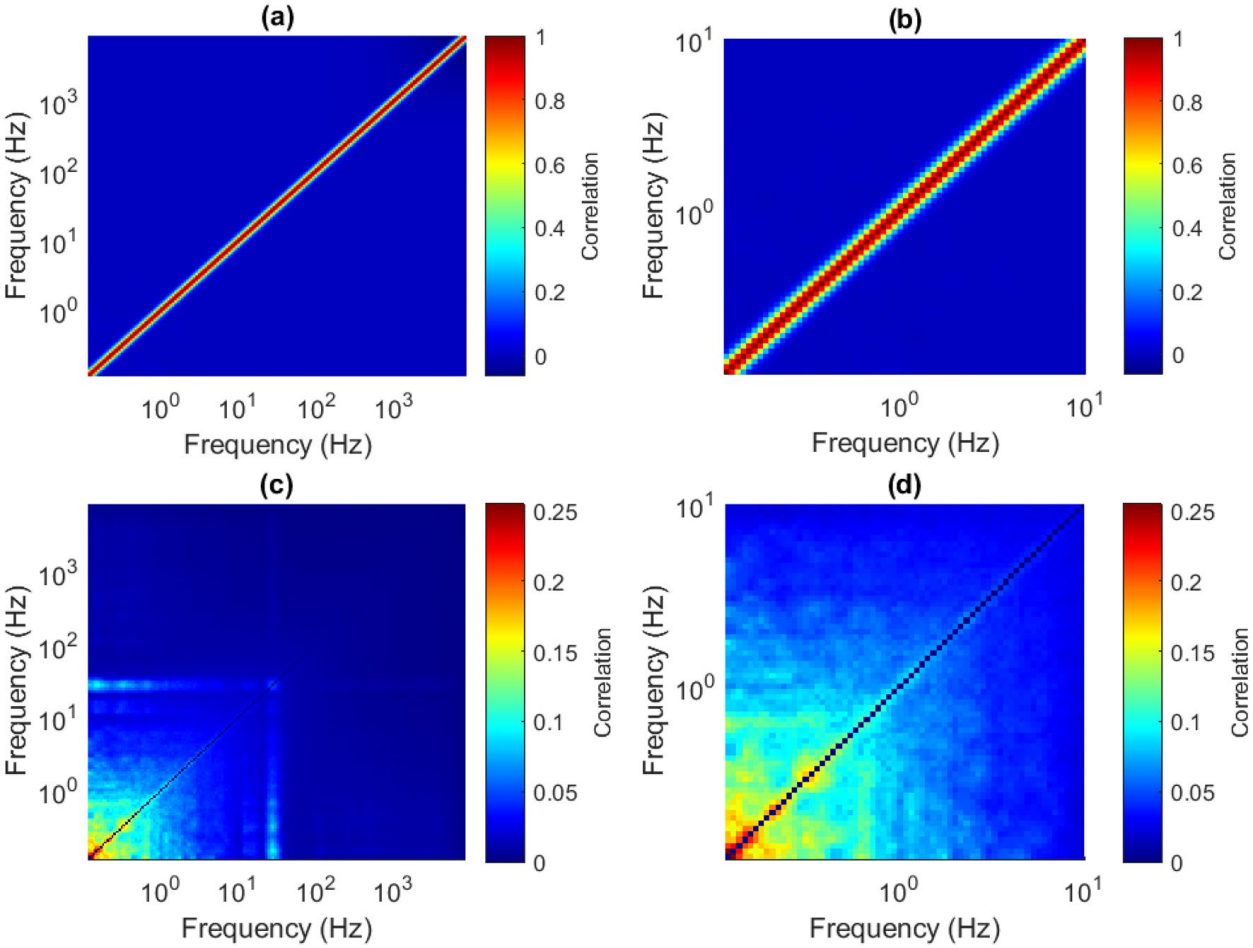
(**a**) Mean ISPCM for white noise process of length 500 s, $L = 1015$. (**b**) Same plot as (**a**), but focused on low–low ISPCs. (**c**) Standard deviation of the ISPCM of the FT phase-randomised WPS for Session. 1, Chan. 1. (**d**) Same plot as (**c**), but focused on low–low ISPCs.

This is particularly the case for low-low scale correlations between significantly overlapping scales. This is because ISPCM elements near the diagonal are most affected by the frequency oversampling (see Fig. 12a,b). Additionally, the variance of the distribution obtained from phase-randomised WPS is generally greater for low-low scale power correlations (Fig. 12c,d). As such, even perfect measured correlations between low-low scales, with significant overlap, may not be significant under the proposed test. As such, the proposed test is extremely conservative in that case, i.e. for values near the diagonal and especially towards the bottom left of the ISPCM. When ISPCs between non-significantly overlapping scales are concerned, the test gives more reliable answers.

The author did not find any obvious unbiased way to eliminate the non-linear distorting effects of the frequency oversampling, the signature of which is shown in the white noise mean ISPCM. As such, simply subtracting it provided a conservative solution to determining significant measured ISPCs. It generally has no effect on ISPCs sufficiently far from the diagonal (Fig. 12a,b), as scales are asymptotically uncorrelated with increased frequency-domain distance. As such, it is included to prevent false positives near the diagonal.

### Validation of the test on synthetic data

To verify that the test works on synthetic data, a set of signals were produced: a double sinusoid, a double pulse, a double chirp, a square wave, an exponential signal, and white noise. Their ISPCMs are produced, tested and discussed. The code for generating and analysing the synthetic data is at^78^. All signals had an overall signal length of 50 s, sampled at 1 kHz. The signals were clipped so that the last value of the signal was within 1% of the 1st value. In calculating the ISPCMs, scales with fewer than 90% data points within the COI were excluded. 250 MC iterations were used for the production of the white-noise ISPCMs, and 150 for the ISPCMs generated from the phase-randomised CWTs. The FDR $\alpha$ was controlled at 0.001 for each test, meaning that 0.1% of the correlations found to be significant are expected to be false positives.

#### Double sinusoid

**Figure 13:**
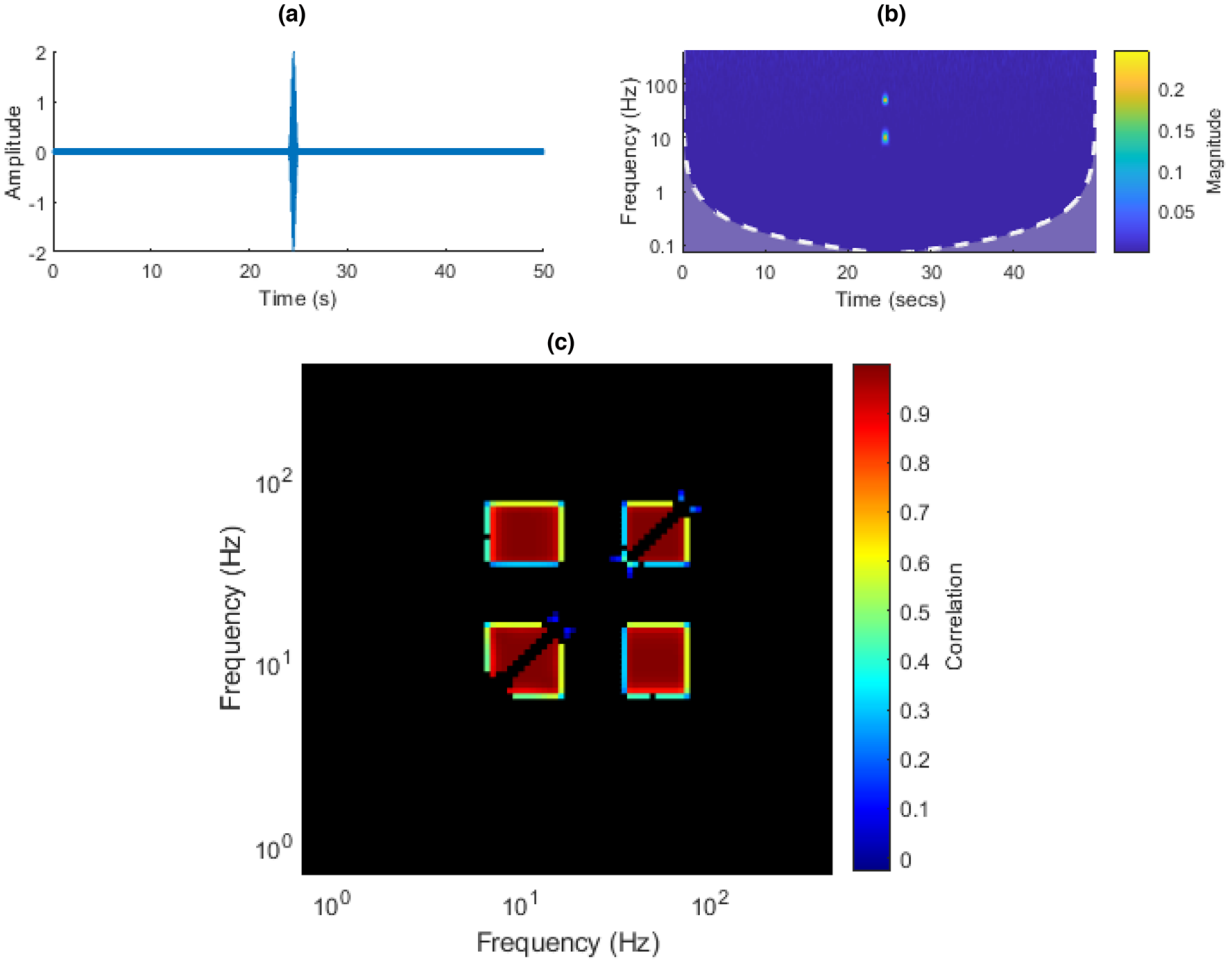
(**a**) Double-sinusoid time domain plot. (**b**) Double-sinusoid CWT. (**c**) Double-sinusoid ISPCM, black elements represent non-statistically significant correlations at an FDR $\alpha$ of 0.001.

In this signal, two sinusoids and low-amplitude Gaussian noise were added together. The entire signal was then enveloped using a Hanning window. The resulting signal can be observed in Fig. 13a,b. The Hanning window was chosen because it has small edge discontinuities and a narrow main lobe. As such it minimized the effects of spurious ISPCs better than other examined windows, e.g. Hamming and Blackman. This was observed in experimentation.

In the double sinusoid signal, one would expect to see four main groupings in the ISPCM. Two should be centered at each of one of the sinusoidal frequencies on the diagonal, where frequency bands are correlated with themselves. The enveloping will distort the sinusoids somewhat, spreading the frequencies. In the ISPCM, this will manifest as a group of frequencies near the sinusoidal frequency being significantly correlated. The other two groupings represent the correlation between the two sinusoidal frequencies, i.e. the groupings not on the diagonal. Some additional minor groupings are expected due to the modulation of the envelopes which can cause some distortion in the ISPCM. However, the conservativeness of the FDR $\alpha$ value seemed to filter these minor groupings out. The main groupings can be observed in Fig. 13c. The first sinusoid’s frequency was 10 Hz, and the second sinusoid’s frequency was 50 Hz.

#### Double pulse

In this signal, two modulated pulses, at different times, were created. Each consisted of a sinusoidal wave at a unique frequency modulated by a Hanning window, with added Gaussian noise.

**Figure 14:**
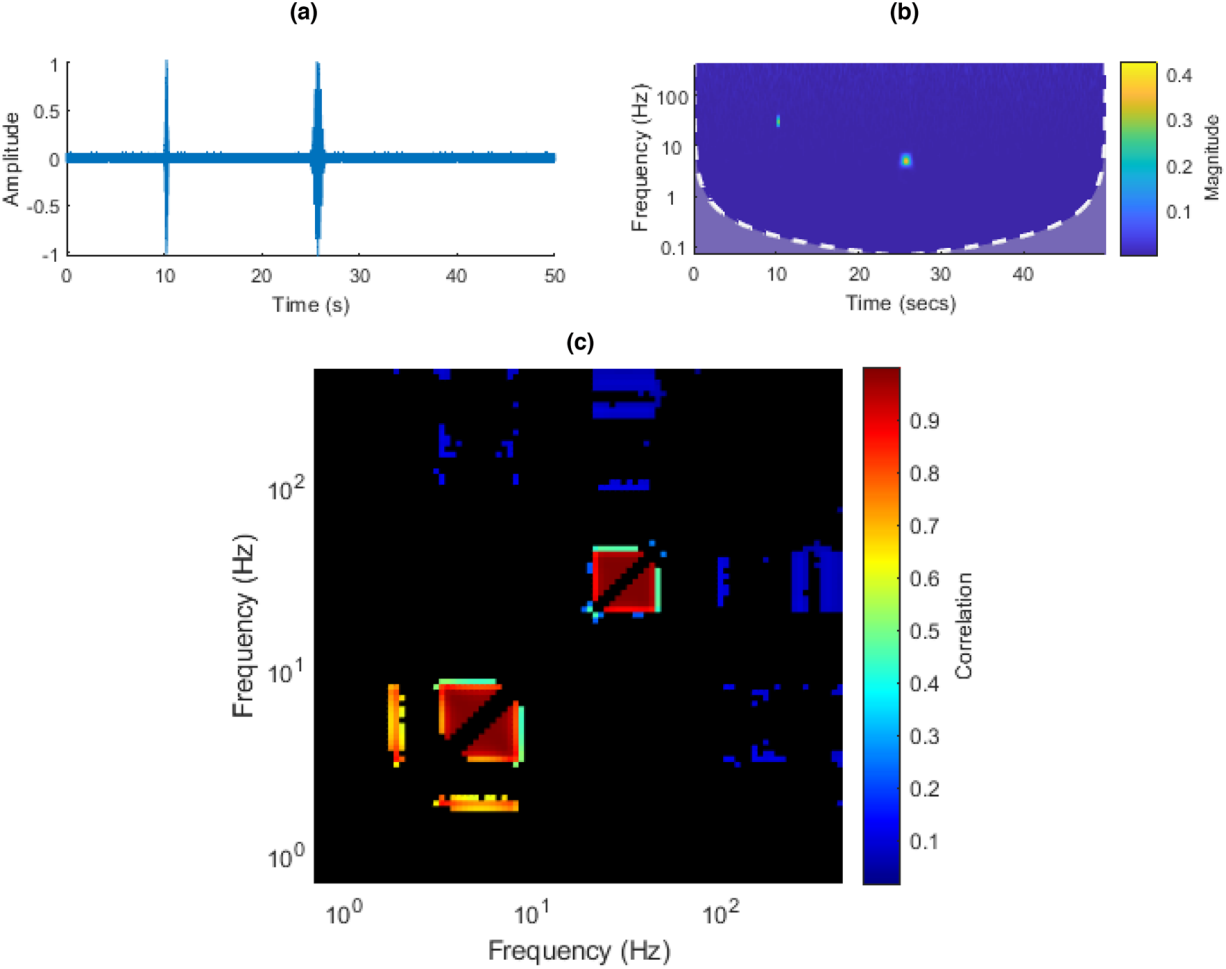
(**a**) Double-pulse time domain plot. (**b**) Double-pulse CWT. (**c**) Double-pulse ISPCM, black elements represent non-statistically significant correlations at an FDR $\alpha$ of 0.001.

In the double pulse signal (Fig. 14a,b), one would expect to see two main groupings in the ISPCM, each centered at one of the carrier frequencies on the diagonal. Some additional minor groupings are expected due to the modulation of the envelopes which can cause some distortion in the ISPCM. These groupings can be observed in Fig. 14c. The first pulse’s carrier frequency is 30 Hz, present between 10 and 10.5 s. The second pulse’s carrier frequency is 5 Hz, present between 25 and 26.5 s.

#### Double chirp

**Figure 15:**
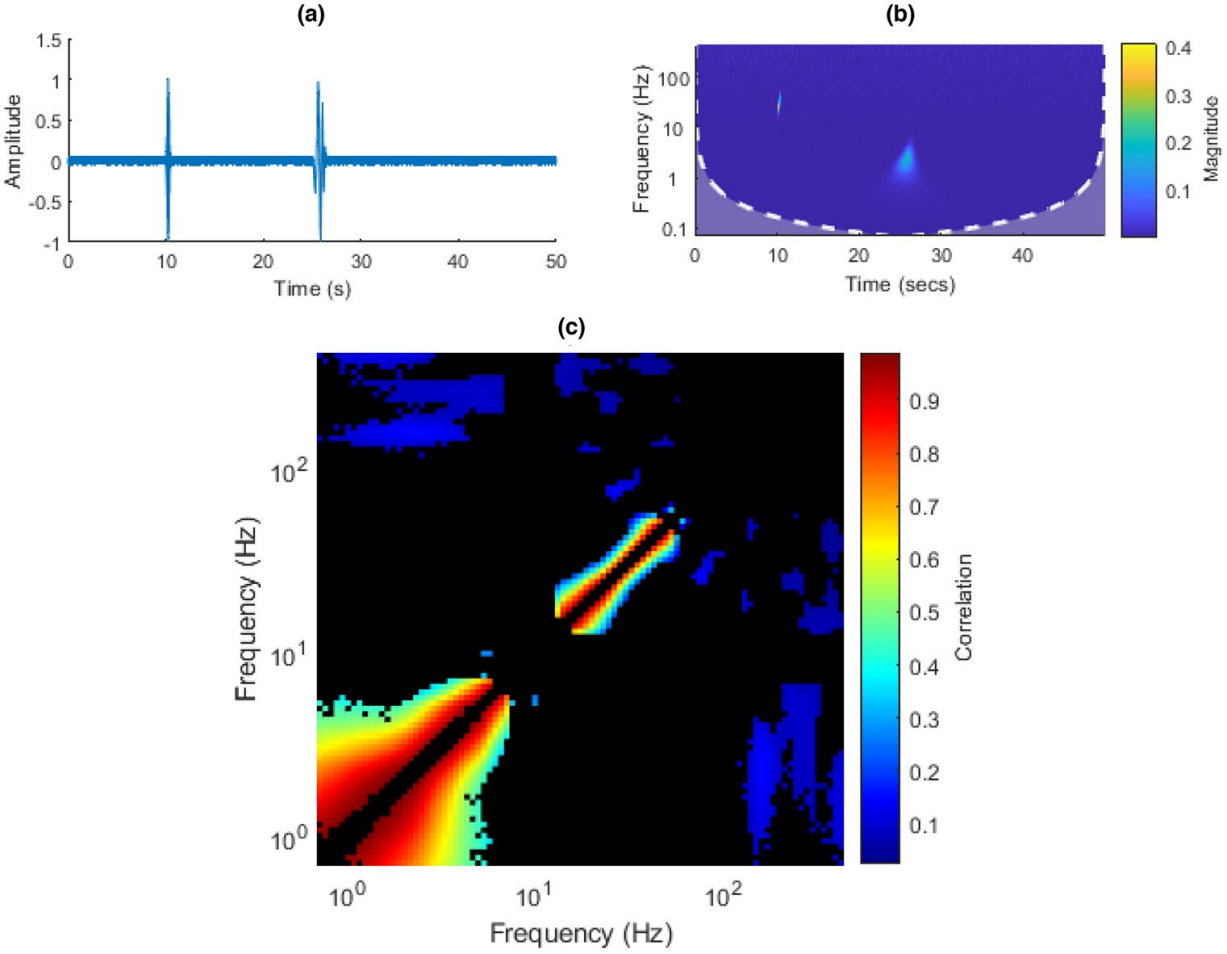
(**a**) Double-chirp time domain plot. (**b**) Double-chirp CWT. (**c**) Double-chirp ISPCM, black elements represent non-statistically significant correlations at an FDR $\alpha$ of 0.001.

In this signal, two enveloped chirps were created. Each consisted of a sinusoidal carrier wave quadratically increasing in frequency, enveloped by a Hanning window, with added Gaussian noise. This can be observed in Fig. 15a,b.

As in the double pulse signal, one would similarly expect to see two main groupings in the ISPCM, each centered at the carrier frequencies. One would expect these groupings to somewhat hug the diagonal, as lower frequencies in the chirp are less correlated to higher frequencies in the chirp than to medium frequencies. This can be observed in Fig. 15c. As with the double pulse, some additional minor groupings are expected. The first chirp’s frequency increased quadratically from 20 to 50 Hz, between 10 and 10.5 s. The second chirp’s frequency similarly increased from 1 to 5 Hz, between 25 and 26.5 s.

#### Square wave

**Figure 16:**
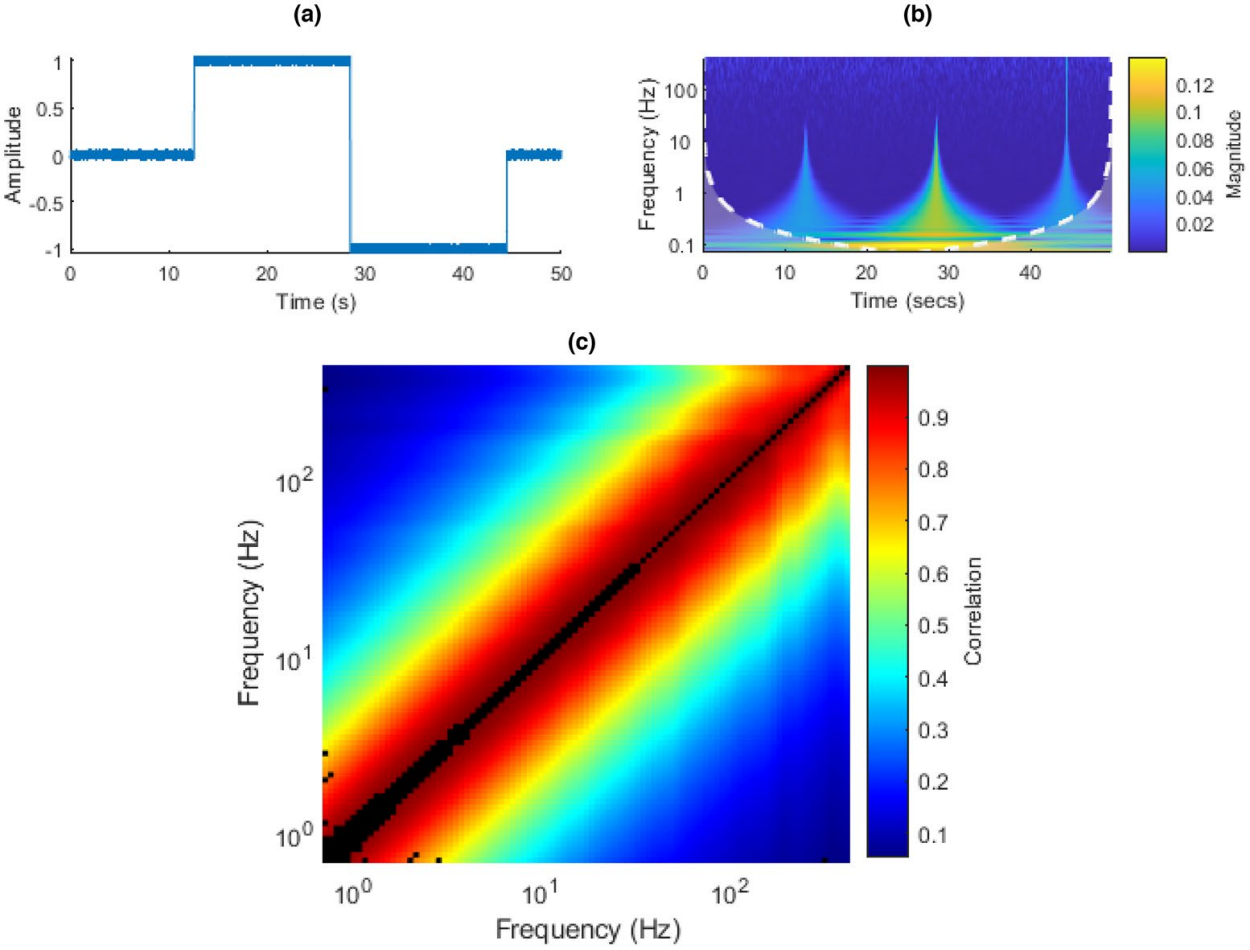
(**a**) Square-wave time domain plot. (**b**) Square-wave CWT. (**c**) Square-wave ISPCM, black elements represent non-statistically significant correlations at an FDR $\alpha$ of 0.001.

The Fourier expansion of a square wave with Gaussian noise is given by a weighted sum of odd harmonics of the central frequency plus noise:

12 $$\begin{aligned} x(t) = \frac{4}{\pi }(\sin (\omega t) + \frac{1}{3} \sin (3 \omega t) + \frac{1}{5} \sin (5 \omega t) + \cdots ) + \epsilon \, (t), \end{aligned}$$

where $\omega$ is $2 \pi f$, where *f* is the central square wave frequency and $\epsilon \, (t)$ is Gaussian noise. The signal can be observed in Fig. 16a,b.

One would expect basically all of the other inter-frequency power correlations to be significant: the signal is highly structured, and the power in one frequency band is highly temporally correlated with that in others. This can be observed in Fig. 16c.

The square wave is a useful signal to demonstrate one of the limitations of the method, which is that it is overly conservative for values near the diagonal, especially at lower frequencies. This was discussed in “Conservatively removing the effects of the frequency-domain oversampling”. This can be observed in how values near the diagonal and especially at lower frequencies are found to not be significant in Fig. 16c.

#### Exponential increase

An exponential signal was considered, where:

13 $$\begin{aligned} x(t) = e^t + \epsilon \, (t), \end{aligned}$$

where $\epsilon (t)$ is Gaussian noise.

This mostly serves to demonstrate the limitations of the proposed method, especially as it relates to clipping. The third condition we gave in “Signal conditions for the use of this test” is not met. This is because there is no segment of interesting length of *x* between some indices $k_1$ and $k_2$, where $1 \le k_1 \ll k_2 \le n$ and $x_{k_1} \approx x_{k_2}$, that can be selected during clipping. This is because, for all $k_1 \ll k_2$, $x_{k_1} \ll x_{k_2}$. As such, running the method as is returned an error, as the length of the clipped signal was too small to run the CWT. Future work should look at understanding the effects of windowing, e.g. in the case of strongly monotonous functions, or of eliminating clipping, on the ISPCM.

#### White noise

**Figure 17:**
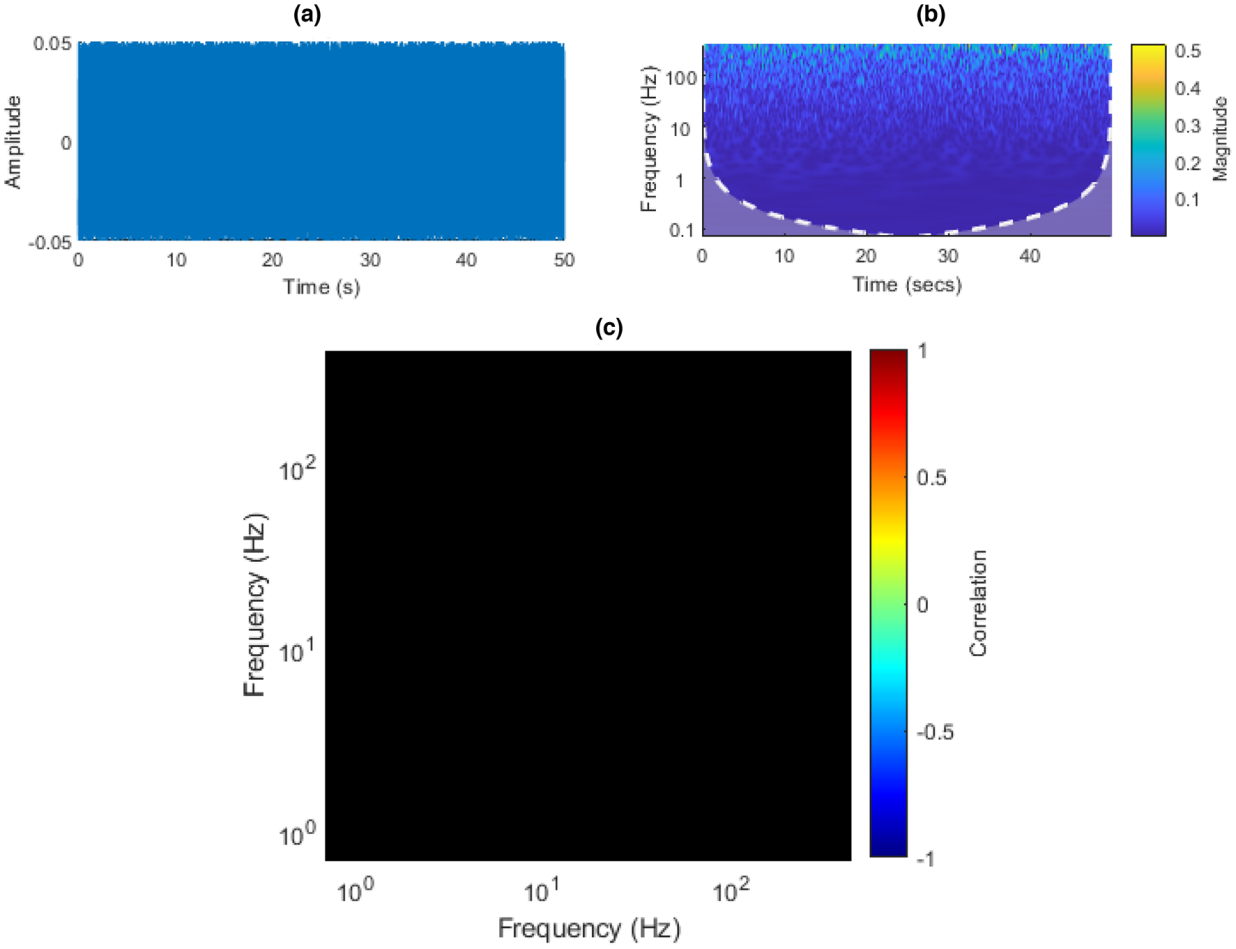
(**a**) White noise time domain plot. (**b**) White noise CWT. (**c**) White noise ISPCM, black elements represent non-statistically significant correlations at an FDR $\alpha$ of 0.001.

We created a white noise signal using MATLAB’s rand function, with the resulting signal shown in Fig. 17a,b. Calculating the statistically significant inter-frequency power correlations in white noise produced a blank matrix where no values were found to be significant. This can be observed in Fig. 17c. This is expected, as one would not expect any significant power correlations between frequency bands in white noise. This is because, by definition, the power in each frequency band in white noise is unstructured.

#### Synthetic data conclusion

The statistical test was validated on synthetic data, and performed as expected. The double pulse and double chirp results demonstrate that abrupt modulation of even simple signal components can produce terms that are somewhat difficult to interpret. These consisted of low-high frequency correlations of significant but small magnitude.

Other than that, the results seem quite intuitive. The significant inter-frequency power correlations that were expected were present. For example, the double-sinusoid signal manifested 4 main clusters as expected in the significant elements of the ISPCM: one centered at each frequency, and their cross-correlations. Similarly, inputting white noise generated no statistically significant ISPCs. Finally, results near the diagonal, especially at lower frequencies, were found to not be significant due to the conservativeness of the test in that circumstance. This is especially visible in the square wave ISPCM.

### Code and full results

The code used throughout this work is available at^77^ and^78^. The full results, including the ISPCMs, the MC-produced null distributions, and the SI-CSD processed neural data time and Fourier domain plots are all available at^77^.

The null distributions for both the FT and IAAFT methods are provided in^77^. For the IAAFT method, the null distribution are only provided for channels 1-20, due to the computational expense in computing them. The code to produce them is available at^77^.

Code to analyse an arbitrary signal is also available at^77,78^.

## Supplementary Information

Supplementary Information.
