# Supplementary material for: The significance of neural inter-frequency power correlations
Source: Sci Rep. 2021 Nov 30;11:23190. doi: 10.1038/s41598-021-02277-0 (PMC8633012; doi:10.1038/s41598-021-02277-0)
Supplement: Supplementary file 1 — Supplementary Information. [file 41598_2021_2277_MOESM1_ESM.pdf]

# Supplemental Material: The Significance of Neural Inter-Frequency Power Correlations

Oscar W. Savolainen, *Student Member, IEEE*

November 20, 2021

## 1 Testing the Normality of the Phase-Randomisation Null Distributions

The vast majority of null distributions produced by FT phase-randomisation were normal. As such, they were compatible with the multiple testing procedure from [1]. The normality was confirmed visually for each analysed recording by using indicator maps of the statistically normal distributions. Fig. 1 shows the indicator map of normal distributions for a small number of randomly selected recordings, as an example.

## 2 Spectral Interpolation Implementation

The original paper on Spectral Interpolation (SI) outlines the general method [4]. In this work, to remove the line noise, we transformed the signal to the Fourier domain using the FFT. We identified the peak line noise frequency at ∼60 Hz. We then spectrally interpolated it and all of its harmonics up to the Nyquist frequency. The interpolated segments had a width of 4 Hz. To perform the SI, we averaged the power in the 3 Hz band from either side of the interpolated segment to calculate the edge values of the interpolated segment. We then fitted a $1^{st}$ order polynomial to between the two edge values. We set the interpolated segments' frequency magnitudes as equal to the fitted line, and randomised the phases. This process is shown in Fig. 2.

We also spectrally interpolated across either one or two sharp components that were often present in the Fourier transforms of the recordings. These components varied in location between ∼2.4-2.8 kHz, and did not correspond to line noise harmonics. These interpolated segments were generally wider than 4 Hz. We also spectrally interpolated the frequency domain between 55 and 65 Hz, due to abnormally large components in this range. We performed no other SI or artifact removal.

## 3 Current Source Density Referencing Implementation

For a review of Current Source Density (CSD) referencing, see [5]. In a Utah array, CSD involves subtracting the average of the four electrodes around the referenced electrode from the recording. In this work, the procedure involved lowpass filtering the referencing signals at 500 Hz. For this we used the MATLAB function `lowpass`.We then averaged the lowpassed data, from the 4 electrodes that formed a square around the referenced electrode, to produce the reference signal. This reference signal was then subtracted from the referenced signal. Some of the electrodes existed on the edge/corner of the Utah array. In these cases, for references we used only the 3/2 electrodes that would have been used had it had 4 neighbours. Examples of the different geographies are shown in Fig. 3 (a). An example of CSD referencing of a neural signal is shown in Fig. 3 (b). The CSD referencing is shown in Fig. 3 (c-d).

The neural medium may be non-capacitive [6]. As such, using only sub-500 Hz components in the reference signal was a somewhat arbitrary choice. However, it had the benefit of preventing high amplitude spikes from affecting the referencing.

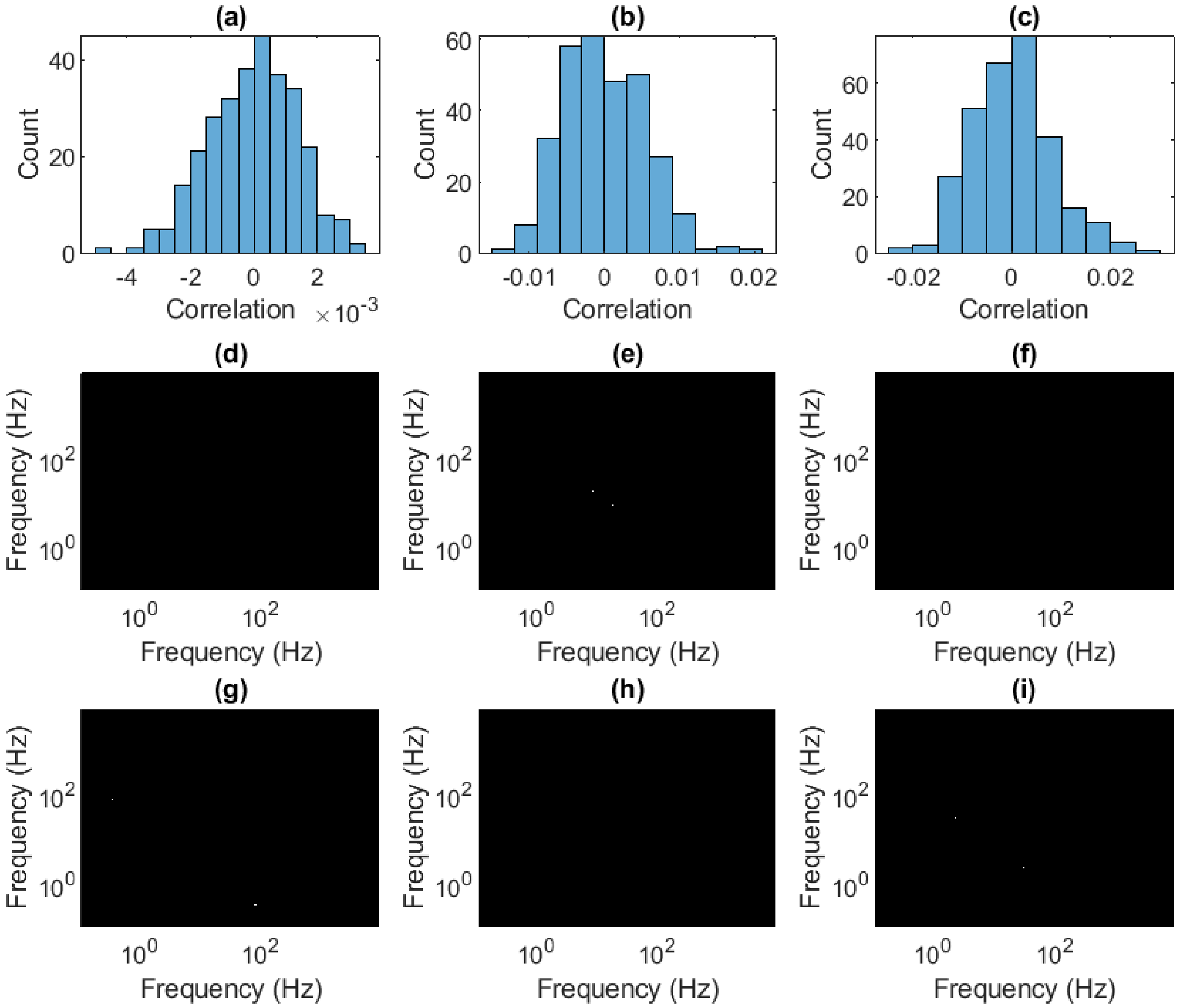


Figure 1: (a-c) Representative examples of $\vec{\phi_{a,b}}$ histograms from Session 1, Chan. 1. (a) Scales 50 and 10. (b) Scales 50 and 80. (c) Scales 50 and 130. (d-g) Indicator maps of double normality-rejected null ISPC $\phi$ distributions for a random selection of recordings. Black points indicate that, out of 10 normality tests, at least 9 failed to reject the null ISPC distribution as non-normal. White points indicate that at least 2 tests found the null ISPC distribution to be non-normal. The FDR was controlled at $\alpha$=0.05 for each test individually using the procedure from [2]. The 10 tests consisted of the Kolmogorov-Smirnov (Limiting form (KS-Lim), Stephens Method (KS-S), Marsaglia Method (KS-M), Lilliefors test (KS-L)), Anderson-Darling (AD), Cramer-Von Mises (CvM), Shapiro-Wilk (SW), Shapiro-Francia (SF), Jarque-Bera (JB), and the D'Agostino and Pearson (DAP) tests [3].

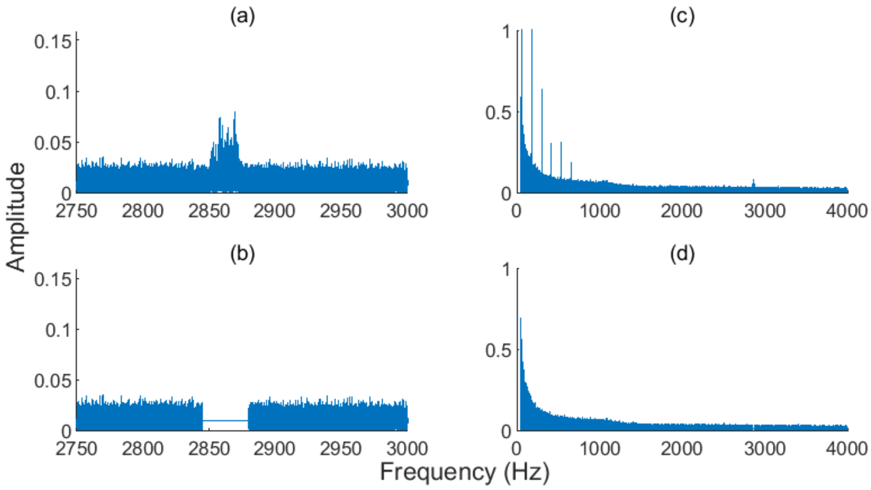


Figure 2: The effect of Spectral Interpolation (SI) on the Fourier domain amplitude of Session 1, Chan. 34, an example channel. (a) A segment of the Fourier domain prior to SI. An unusual, non-line noise component is observable. (b) The same segment post-SI, with the component removed. (c) The Fourier Spectrum between 0 and 4 kHz, without SI, where line-noise harmonics are clearly visible. (d) The Fourier Spectrum between 0 and 4 kHz, post-SI.

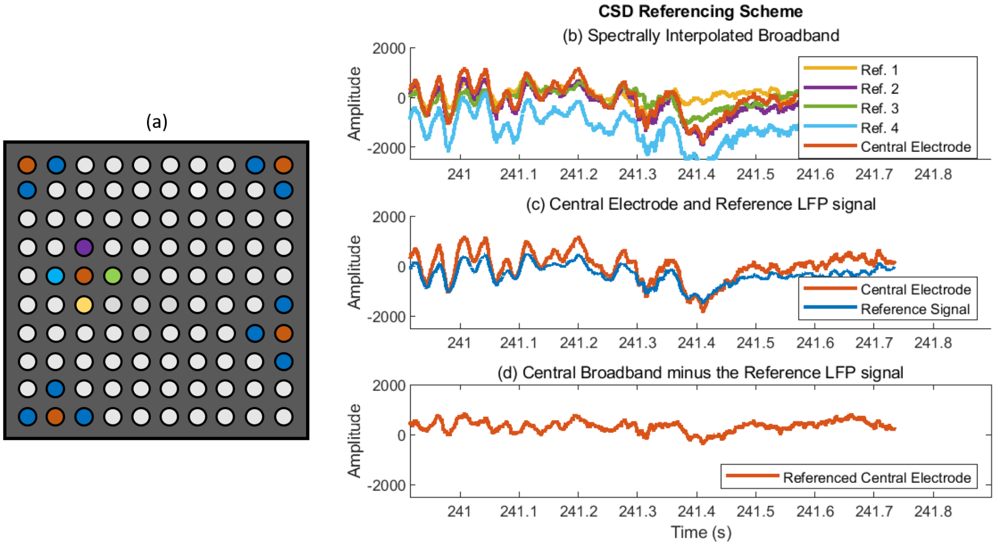


Figure 3: Current Source Density (CSD) Referencing Scheme. (a) A few examples of CSD referencing of Utah Array electrodes. Orange electrodes indicate examples of referenced electrodes. The adjacent dark blue electrodes indicate the electrodes whose lowpassed signals are averaged across to create the reference signal for the given orange electrode. Purple, blue, yellow and green indicate the electrodes used for referencing their central orange electrode. (b) The lowpassed signals from the purple, blue, yellow and green electrodes in (a), for the central electrode Session 1, Chan. 13. (c) The dark blue plot indicates the reference signal, equal to the average of Ref. (1-4) from (b). (d) The referenced signal: the central electrode signal minus the reference signal.

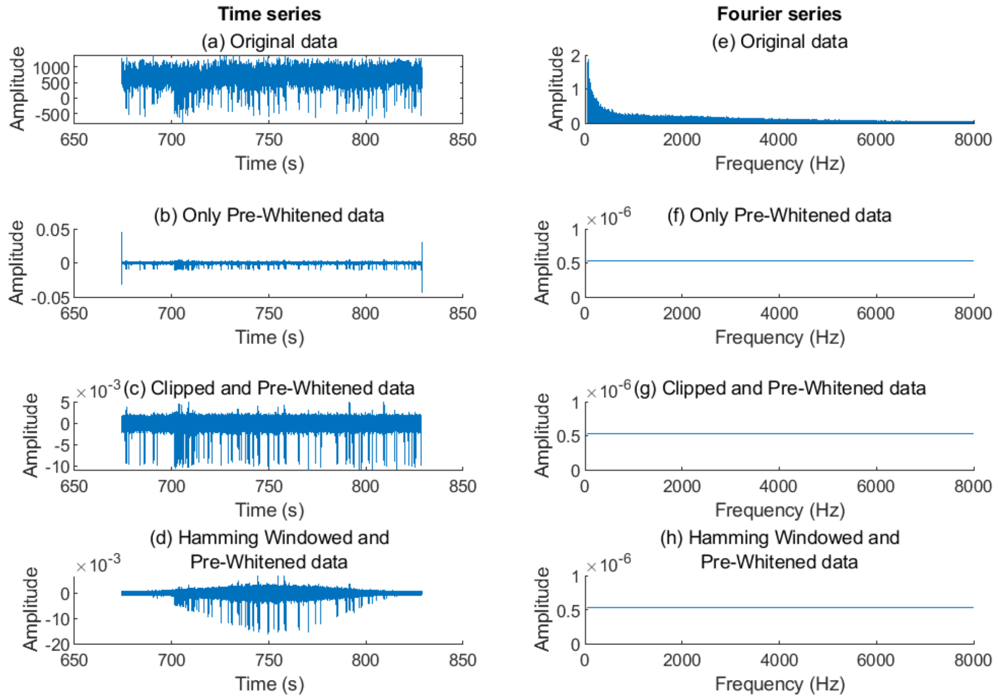


Figure 4: Time and Fourier transform for the neural recording from Session 1, Chan. 1 with various levels of processing. (a, e) The original neural signal. (b, f) The effects of only pre-whitening. (c, g) The effects of clipping and then pre-whitening. (d, h) The effects of applying a Hamming Window and then pre-whitening.

# 4 Clipping Implementation

For the final pre-processing step, the CSD-referenced data was clipped. First, we removed samples $x_{n-k}, \dots, x_n$ so that $k$ was minimised while ensuring that $x_{n-k-1}$ was within 1% of $x_1$. On average, this removed around 50 time steps from the end of the signal, and so had a negligible effect on the signal lengths. Then the signal was zero-meaned. Clipping the end of $\vec{x}$ and zero-meaning aided the assumption of periodicity in the Fourier transform of each scale to hold. The main consequence of this is that the power at every scale was closer to zero at the beginning and end of the zero-centered clipped sequence. This somewhat mitigated the effects of cyclic discontinuities during phase-randomisation of each scale. This is not strictly necessary, as cyclic discontinuities, if they are part of the intra-scale signal, are part of the intra-scale signal. As such it is not obvious that they, or their downstream effects on $\vec{\phi}_{a,b}$ and thus the null distribution, should be mitigated. However, in this work they were somewhat mitigated, and in doing so clipping was preferable to using a classical window, which would have distorted the normalisation of the ISPC. This is because windowing produces smaller standard deviation at the edges of the signal. This can be observed in Fig. 4 (d).

Generally, the effects of clipping, pre-whitening, and using a Hamming window prior to pre-whitening on the time and Fourier transform of a neural signal are shown in Fig. 4. In Fig. 4, pre-whitening is used to demonstrate the effects of cyclic discontinuities. E.g., Fig. 4 (b) shows that cyclic discontinuities can introduce strong artificial high frequency components at the beginning and end of the signal. In phase-randomisation, these artificial components are spread throughout the signal. Since this is unwanted, we need some way to suppress cyclic discontinuities, e.g. clipping or windowing.

It is not necessary for the first index of the clipped segment to be the first value of the original signal. It can be any arbitrary index. Generally, the clipped segment needs to be of sufficient length, between indices $k_1, k_2$ such that $x_{k_1} \approx x_{k_2}$ and $1 \leq k_1 \ll k_2 \leq n$. However, in this work the first value of the whole signal was used as the first value of the clipped segment, i.e. $k_1 = 1$.

# 5 Pseudo-Random Number Generation for Monte Carlo White Noise Processes

A subtle point worth mentioning is that the MATLAB `rand` function uses Mersenne Twisters to produce pseudo-random numbers. The use of Mersenne Twister's in MC simulations has been criticised [7]. As such, the authors used the PCG family of RNGs [8] to produce pseudo-random numbers for the white noise processes. The state seed was set as 1 for all cases, and the sequence seed was set as $n + v$. We used a MC sequence length of either $n = F_s \times 350\,s = 8544922$, $n = F_s \times 400\,s = 9765625$, or $n = F_s \times 500\,s = 12207031$ samples. The C code from [8] was adapted to create the 32-bit white noise processes and save them to '.txt' files. These were then accessed by the appropriate MATLAB functions. In MATLAB, the white noise processes were then processed as described in Section 4.4.1 and Fig. 10 in the main text.

# References


[1] T. T. Cai and W. Liu, "Large-scale multiple testing of correlations," Journal of the American Statistical Association, vol. 111, no. 513, pp. 229–240, 2016.

[2] Y. Benjamini and D. Yekutieli, "The control of the false discovery rate in multiple testing under dependency," Annals of statistics, pp. 1165–1188, 2001.

[3] D. Groppe, "Fdr bh," https://www.mathworks.com/matlabcentral/fileexchange/27418-fdr_bh, MATLAB Central File Exchange, 2020, accessed: 2020-12-14.

[4] S. Leske and S. S. Dalal, "Reducing power line noise in eeg and meg data via spectrum interpolation," NeuroImage, vol. 189, pp. 763–776, 2019.

[5] U. Mitzdorf, "Current source-density method and application in cat cerebral cortex: investigation of evoked potentials and eeg phenomena," Physiological reviews, vol. 65, no. 1, pp. 37–100, 1985.

[6] Y. Kajikawa and C. E. Schroeder, "How local is the local field potential?" Neuron, vol. 72, no. 5, pp. 847–858, 2011.

[7] S. Vigna, "It is high time we let go of the mersenne twister," arXiv preprint arXiv:1910.06437, 2019.

[8] M. E. O'Neill, "Pcg: A family of simple fast space-efficient statistically good algorithms for random number generation," Harvey Mudd College, Claremont, CA, Tech. Rep. HMC-CS-2014-0905, Sep. 2014.
